# N-hydroxypipecolic acid primes plants for enhanced microbial pattern-induced responses

**DOI:** 10.3389/fpls.2023.1217771

**Published:** 2023-08-14

**Authors:** Marie Löwe, Katharina Jürgens, Tatyana Zeier, Michael Hartmann, Katrin Gruner, Sylvia Müller, Ipek Yildiz, Mona Perrar, Jürgen Zeier

**Affiliations:** ^1^ Institute for Molecular Ecophysiology of Plants, Department of Biology, Heinrich Heine University, Düsseldorf, Germany; ^2^ Cluster of Excellence on Plant Sciences (CEPLAS), Heinrich Heine University, Düsseldorf, Germany

**Keywords:** N-hydroxypipecolic acid, priming, flagellin response, systemic acquired resistance, salicylic acid, *Arabidopsis thaliana*, plant immunity

## Abstract

The bacterial elicitor flagellin induces a battery of immune responses in plants. However, the rates and intensities by which metabolically-related defenses develop upon flagellin-sensing are comparatively moderate. We report here that the systemic acquired resistance (SAR) inducer N-hydroxypipecolic acid (NHP) primes *Arabidopsis thaliana* plants for strongly enhanced metabolic and transcriptional responses to treatment by flg22, an elicitor-active peptide fragment of flagellin. While NHP powerfully activated priming of the flg22-induced accumulation of the phytoalexin camalexin, biosynthesis of the stress hormone salicylic acid (SA), generation of the NHP biosynthetic precursor pipecolic acid (Pip), and accumulation of the stress-inducible lipids γ-tocopherol and stigmasterol, it more modestly primed for the flg22-triggered generation of aromatic and branched-chain amino acids, and expression of *FLG22-INDUCED RECEPTOR-KINASE1*. The characterization of the biochemical and immune phenotypes of a set of different Arabidopsis single and double mutants impaired in NHP and/or SA biosynthesis indicates that, during earlier phases of the basal immune response of naïve plants to *Pseudomonas syringae* infection, NHP and SA mutually promote their biosynthesis and additively enhance camalexin formation, while SA prevents extraordinarily high NHP levels in later interaction periods. Moreover, SA and NHP additively contribute to Arabidopsis basal immunity to bacterial and oomycete infection, as well as to the flagellin-induced acquired resistance response that is locally observed in plant tissue exposed to exogenous flg22. Our data reveal mechanistic similarities and differences between the activation modes of flagellin-triggered acquired resistance in local tissue and the SAR state that is systemically induced in plants upon pathogen attack. They also corroborate that the NHP precursor Pip has no independent immune-related activity.

## Introduction

Pattern-triggered immunity (PTI) is an important first line of inducible plant defense against attack by pathogenic microbes (DeFalco and Zipfel, 2021). PTI is based on the recognition of molecular patterns that either represent conserved microbial structures (pathogen- or microbe-associated molecular patterns [PAMPs, MAMPs]) or products released by the plant host after tissue damage (damage-associated molecular patterns [DAMPs]) (Lee et al., 2021). Well-characterized PAMPs from bacteria include flagellin, lipopolysaccharides (LPS) and elongation factor Tu (EF-Tu), while chitin, β-1,3-glucan and ergosterol constitute classical fungal PAMPs (Boutrot and Zipfel, 2017). Flagellin is the most prominent protein subunit of the eubacterial flagellum, which functions as a motility organelle (Haiko and Westerlund-Wikström, 2013). In *Arabidopsis thaliana*, flagellin is perceived by leucine-rich repeat (LRR) domains of the plasma membrane-resident receptor kinase FLAGELLIN-SENSING 2 (FLS2) (Gómez-Gómez and Boller, 2000; Chinchilla et al., 2006). The elicitor-active domain of flagellin is situated in the N-terminal region of the protein, and a peptide corresponding to a highly conserved 22 amino acid stretch of the flagellin protein (flg22) functions as a potent elicitor of plant defense responses (Felix et al., 1999).

Plants activate a series of signaling events and defense responses at the cellular, tissue and organismal levels upon molecular pattern recognition. Flagellin perception results in H^+^- and Ca^2+^-influxes into the cytoplasm, a transient H_2_O_2_-burst, and activation of mitogen-activated protein kinase (MAPK) cascades. Moreover, it induces ethylene biosynthesis, increases expression of pathogenesis-related (PR) genes, and triggers callose deposition to the cell wall (Felix et al., 1999; Gómez-Gómez et al., 1999; Asai et al., 2002; Boller and Felix, 2009). In addition, several defense-related metabolic changes occur in flagellin-exposed plants that are also observed in response to challenge by pathogenic bacteria. In Arabidopsis leaves, flagellin perception induces the biosyntheses of the defense hormone salicylic acid (SA) and the phytoalexin camalexin (Mishina and Zeier, 2007; Tsuda et al., 2008; Frerigmann et al., 2016; Zhao et al., 2021). Flagellin sensing also triggers accumulation of the non-protein amino acid pipecolic acid, the unsaturated sterol stigmasterol, and the vitamin E variant γ-tocopherol (Griebel and Zeier, 2010; Návarová et al., 2012; Stahl et al., 2019).

The flagellin-induced activation of plant defenses during a compatible plant-bacterial interaction significantly contributes to plant basal immunity. This is exemplified by the increased susceptibility of *FLS2*-defective and thus flagellin-insensitive Arabidopsis mutants to infection by virulent *Pseudomonas syringae* strains (Zipfel et al., 2004). Plant basal immunity to compatible bio- and hemibiotrophic pathogens largely depends on a functional SA signaling pathway (Thomma et al., 1998; Nawrath and Métraux, 1999; Dewdney et al., 2000). The more recently employed terminology designates basal immunity also as PTI (Boller and Felix, 2009). However, the contribution of flagellin sensing to PTI within a progressing plant-bacterial interaction (basal immunity) must be distinguished from the enhanced state of immunity that plants acquire upon exogenous (pre-)treatment with flagellin (Zipfel et al., 2004). Flagellin-induced acquired resistance is usually assayed locally, i.e., in the flg22-pretreated tissue (Zipfel et al., 2004; Tsuda et al., 2009). Interestingly, a localized flg22-treatment of leaf tissue can also increase pathogen resistance in distant, non-treated leaves. This systemic response to flagellin mechanistically resemble systemic acquired resistance (SAR) (Mishina and Zeier, 2007).

SAR is commonly defined as a plant response that is induced by a localized leaf inoculation with a pathogen and results in enhanced, broad-spectrum immunity of distantly located leaves (Sticher et al., 1997; Shah and Zeier, 2013; Vlot et al., 2021; Zeier, 2021). This systemic immunization is associated with a strong transcriptional response in the distant tissue that includes the up- and down-regulation of several thousand genes (Bernsdorff et al., 2016). SAR establishment is triggered by the L-Lys-derived, immune-active metabolite N-hydroxypipecolic acid (NHP), which accumulates in both inoculated and distant leaves of a pathogen-attacked plant (Hartmann et al., 2018). NHP is synthesized in response to pathogen inoculation by a biochemical sequence that involves the N-hydroxylation of the non-protein amino acid pipecolic acid (Pip) by FLAVIN-DEPENDENT MONOOXYGENASE1 (FMO1) as a final step (Chen et al., 2018; Hartmann et al., 2018). The NHP biosynthetic precursor Pip, which itself strongly accumulates systemically in Arabidopsis and other plants upon biotic attack (Návarová et al., 2012; Hartmann and Zeier, 2018; Schnake et al., 2020), is biosynthesized by α-transamination of L-Lys via AGD2-LIKE DEFENSE RESPONSE PROTEIN1 (ALD1), and subsequent reduction of the resulting dehydropipecolic acid intermediates by the reductase SAR-DEFICIENT4 (SARD4) (Návarová et al., 2012; Ding et al., 2016; Hartmann et al., 2017).

Accumulation of NHP in plants as a consequence of pathogen attack is indispensable for the biological, pathogen-triggered induction of SAR. In addition, exogenous application of NHP to plants via soil application or treatment of single leaves is sufficient to trigger a strong immune response systemically in the Arabidopsis leaf rosette that closely resembles biologically-induced SAR, both at the resistance and the transcriptional levels (Návarová et al., 2012; Bernsdorff et al., 2016; Chen et al., 2018; Hartmann et al., 2018; Yildiz et al., 2021; Yildiz et al., 2023). Notably, Arabidopsis mutants unable to accumulate SA because of defects in key SA biosynthetic genes such as *ISOCHORISMATE SYNTHASE1* (*ICS1*) or *avrPphB SUSCEPTIBLE3* (*PBS3*) only induce modest NHP-triggered and biological SAR responses (Hartmann et al., 2018; Yildiz et al., 2021). These and other findings demonstrate that the NHP and SA immune pathways positively interact to activate SAR (Zeier, 2021). In addition, the SA receptor NON-EXPRESSOR OF PR GENES1 (NPR1) and TGA transcription factors act downstream of NHP in the induction of SAR and the SAR-associated transcriptional response (Yildiz et al., 2021; Yildiz et al., 2023). Remarkably, for the termination of SAR, the immune-active metabolites NHP and SA are simultaneously glucosylated by the same glycosyltransferase and thus inactivated in concert (Bauer et al., 2021; Cai et al., 2021; Holmes et al., 2021; Mohnike et al., 2021; Zeier, 2021).

The second line of inducible defense at pathogen inoculation sites is termed effector-triggered immunity (ETI). ETI results in the rapid activation of defense responses, is generally associated with a hypersensitive cell death response (HR), and provides effective protection against attempted invasion by incompatible pathogens (Thordal-Christensen, 2020). By comparison, the PTI-related responses that are associated with basal immunity are quantitatively moderate and not able to entirely prohibit compatible pathogen infection. SAR activation by a first localized pathogen inoculation provides a powerful solution for this dilemma, because the SAR state systemically primes plants for a timely and boosted response to compatible microbial challenge, which consequently results in increased immunity at the whole plant level (Jung et al., 2009; Návarová et al., 2012; Conrath et al., 2015). Analysis of Arabidopsis mutants for their capacity to systemically establish a primed state upon leaf pathogen inoculation demonstrate that NHP functions as a decisive signal for SAR-associated defense priming, while SA has an amplifying role in this process (Návarová et al., 2012; Bernsdorff et al., 2016; Hartmann et al., 2018). Consistently, exogenous treatment with NHP proved sufficient to systemically trigger a primed state in Arabidopsis (Yildiz et al., 2021). Plants with activated SAR as a consequence of either an inducing pathogen inoculation or pre-treatment with NHP show a strongly enhanced capacity to trigger metabolic defense reactions in response to a challenge attack by compatible *P. syringae* (Bernsdorff et al., 2016; Yildiz et al., 2021).

We report here that NHP primes plants for a defined pattern-triggered response – the response to bacterial flagellin. Pre-treatment of plants with NHP resulted in a strongly boosted activation of several metabolic defense pathways observed in flg22-exposed leaves, and also primed plants for enhanced expression of flg22-inducible genes. Our data show that the NHP and SA signalling pathways function additively in plant basal resistance to bacterial and oomycete challenge, as well as in early camalexin accumulation. They further indicate that SA accumulation enhances NHP biosynthesis in an early stage of a compatible plant-bacterial interaction, while NHP biosynthesis augments SA production. In later infection stages, however, SA moderates the accumulation of NHP. Further, NHP and SA also additively contribute to the immune response triggered in flg22-treated leaf tissue. Our study reveals mechanistic overlap but also differences between the locally induced flagellin-acquired resistance response and SAR induced systemically by pathogen inoculation. It further corroborates the function of Pip as a per se immune-inactive precursor of its direct derivate, the FMO1-generated and SAR-inducing hormone NHP.

## Materials and methods

### Plant material and cultivation

The cultivation of Arabidopsis (*Arabidopsis thaliana*) plants was conducted as described previously (Hartmann et al., 2018). The plants were grown individually in pots containing a mixture of soil (Substrat BP3; Klasmann-Deilmann), vermiculite, and sand (8:1:1) in a growth chamber with a 10-h-day (9 _AM_ to 7 _PM_)/14-h-night cycle, a photon flux density of 100 μmol m^-2^ s^-1^ during the day, a relative humidity of 60%, and 21°C day and 18°C night temperatures, respectively. Experiments were performed with 5-week-old plants.

The following Arabidopsis lines were used: Col-0 [Nottingham Arabidopsis Stock Centre (NASC) ID: N1092], *sid2* (*sid2-1*; Nawrath and Métraux, 1999), *ald1* (Salk_007673; Návarová et al., 2012), *fmo1* (Salk_026163; Mishina and Zeier, 2006), *sid2 ald1* (*sid2-1 ald1*; Bernsdorff et al., 2016), *sid2 fmo1* (*sid2-1 fmo1*; this study), *ald1 fmo1* (this study), *fls2* (*fls2c*; SAIL_691C4; Zipfel et al., 2004), *pad4* (*pad4-1*; N3806), *eds1* (*eds1-2*; Bartsch et al., 2006), *npr1* (*npr1-3*; N3802), *mpk3* (*mpk3-1*; Salk_151594; Wang et al., 2018a), and *mpk6* (*mpk6-2*; Salk_073907; Wang et al., 2018a).

The *sid2 fmo1* double mutant was generated by crossing *sid2-1* (female parent) and *fmo1* (male parent) single mutants. F1 seeds were collected from fertilized siliques, planted, and flowering F1 plants self-fertilized. The resulting F2 plants were screened for homozygosity of the *fmo1* (Salk_026163) T-DNA insertion using PCR-based genotyping with gene specific and T-DNA left border (LBb1.3) primers (**Supplementary Table 1**; **Supplementary Figure 4**; Mishina and Zeier, 2006; O’Malley et al., 2015). Plants homozygous for the *fmo1* genotype were examined for the presence of the *sid2-1* genotype by verifying the SA-induction-deficiency by GC-MS analysis of *P. syringae*-inoculated plants as described below. Similarly, the *ald1 fmo1* double mutant was generated by crossing *ald1* (female parent) and *fmo1* (male parent) single mutants. The homozygosity of *fmo1* and *ald1* genotypes was confirmed by PCR-based analyses using gene specific primers and the LBb1.3 T-DNA left border primer (**Supplementary Table 1**; **Supplementary Figure 4**; Mishina and Zeier, 2006; Návarová et al., 2012; O’Malley et al., 2015; Bernsdorff et al., 2016).

### Cultivation of Pseudomonas syringae, plant inoculation and bacterial growth assays

For bacterial inoculations, *Pseudomonas syringae* pv. *maculicola* strain ES4326 (*Psm*), *Psm* expressing the luxCDABE operon from *Photorhabdus luminescens* (*Psm lux*), *P. syringae* pv. *tomato* DC3000 (*Pst*) expressing luxCDABE (*Pst lux*), *Psm* expressing AvrRpm1 (*Psm avrRpm1*), and *Pst* expressing AvrRpt2 (*Pst avrRpt2*) were cultivated at 28°C in King’s B medium with the appropriate antibiotics as described (Fan et al., 2008; Tsuda et al., 2009; Bernsdorff et al., 2016; Gruner et al., 2018). For plant inoculation, bacterial suspensions obtained from overnight cultures were washed with 10 mM MgCl_2_ and diluted to different final optical densities at 600 nm (OD_600_). The bacterial suspensions were then carefully infiltrated into Arabidopsis rosette leaves using needleless syringes in the morning between 10_AM_ and 12_PM_.

For the determination of metabolite accumulation upon *Psm* challenge, suspensions of OD_600 =_ 0.005 were infiltrated into three rosette leaves of 5-week-old Arabidopsis plants. As a control treatment, a mock-infiltration with 10 mM MgCl_2_ solution was performed. The treated leaves were harvested at 12, 24 or 48 h after treatment, fresh weights (FW) determined and the leaf samples shock-frozen in liquid nitrogen. Each replicate sample consisted of six leaves from two different plants. Four to five replicate samples were analyzed in each experiment.

For the bacterial growth assays, the *Psm lux*, *Pst lux*, *Psm avrRpm1* and *Pst avrRpt2* strains were diluted to OD_600 =_ 0.001 and the suspensions infiltrated into three Arabidopsis rosette leaves. The compatible, bioluminescent *Psm lux* and *Pst lux* strains were used to assess basal resistance (Fan et al., 2008; Gruner et al., 2018). Bacterial numbers were determined 2.5 days post inoculation (dpi) by measuring the bioluminescence of leaf discs from the inoculated leaves (d = 12 mm, one disc per inoculated leaf) with a Sirius FB12 luminometer (Berthold Detection Systems, http://www.titertek-berthold.com). The bacterial numbers were expressed as relative light units (rlu) per cm^2^ leaf area. At least 15 replicate leaf samples were assayed for one genotype and/or treatment. To assess ETI-related resistance, three leaves per plant were infiltrated with *Psm avrRpm1* or *Pst avrRpt2.* The infiltrated leaves were harvested at 3 dpi, and three leaf discs from the three infiltrated leaves per plant were homogenized in 1 ml 10 mM MgCl_2_. Appropriate dilutions (in 10 mM MgCl_2_) were plated on King’s B medium containing rifampicin (50 µg l^-1^), and the numbers of developing colonies on plates were quantified two days after incubating them at 28°C (Zeier et al., 2004). The bacterial numbers were expressed as colony-forming units (cfu) per cm^2^ leaf area. At least 9 replicate samples were analyzed for one genotype.

### Inoculation of Hyaloperonospora arabidopsidis and growth assay

The protocol for inoculation of Arabidopsis with *Hyaloperonospora arabidopsidis* (*Hpa*) isolate Noco2 and the associated disease scoring procedure was described previously in detail (Hartmann et al., 2018). Briefly, the rosette leaves were spray-inoculated with a suspension of 5×10^4^ sporangia per ml of H_2_O. The inoculated plants were then maintained for 5 days on trays sealed with a transparent lid under the above-mentioned growth conditions. Leaves were harvested, stained with Trypan blue and destained with chloral hydrate solution. Photographic images of leaves were captured with a Canon EOS 6D DSLR camera, and the digital images analyzed using the ImageJ software to determine the length of intercellular hyphae (IH) per cm^2^ leaf area.

### Flagellin-induced acquired resistance and systemic acquired resistance

The local flagellin-induced acquired resistance response was generally determined by co-applying flg22 peptide (Felix et al., 1999; synthesized by Mimotopes; http://www.mimotopes.com/) with compatible *Psm lux* to leaves. More specifically, suspensions of *Psm lux* (OD = 0.001) containing or lacking 1 µM of flg22 were infiltrated into three leaves of a given plant and bacterial numbers assessed 2.5 days later in the same leaves via the determination of bacterial bioluminescence as described above. In addition, a pre-application procedure was performed, whereby three leaves of a given plant were syringe-infiltrated with an aqueous solution of 1 µM flg22 or with water as a control treatment. One day later, the same leaves were inoculated with *Psm lux* and bacterial numbers assessed 2.5 days later. 15 to 18 replicate leaf samples were assayed per genotype and treatment.

To assess SAR, three lower rosette leaves of a plant were inoculated with *Psm* (OD_600_ = 0.005) or mock-infiltrated with 10 mM MgCl_2_, and three upper leaves challenge-inoculated with *Psm lux* (OD_600_ = 0.001) two days later. The numbers of *Psm lux* were assessed 2.5 days after the challenge inoculation via bioluminescence determination (see above; Gruner et al., 2018). At least 15 replicate leaf samples were assayed for one genotype and treatment.

### Exogenous treatments with NHP or Pip to asses priming of flg22 responses

Exogenous plant treatments were performed with an aqueous, 1 mM N-hydroxypipecolic acid (NHP; Hartmann et al., 2018) or a 1 mM pipecolic acid (Pip; Sigma-Aldrich S47167; Návarová et al., 2012) solution. Therefore, 10 ml of NHP (Pip) solution or 10 ml of water (control treatment) was pipetted onto the soil of individually cultivated Arabidopsis Col-0 plants. One day later, three rosette leaves were infiltrated with 1 µM flg22 solution or mock-treated with water. The leaves of another set of plants were not treated at all after the NHP (Pip) treatment. The leaves were harvested at 8, 24 or 48 h after the flg22- or control-treatments and frozen in liquid nitrogen for the determination of metabolite contents. The transcript levels of defense-related genes were assessed at 8 h post leaf treatment. Replicate leaf samples consisted of six leaves from two different plants, and three replicate samples per treatment and time point were analyzed.

### Determination of leaf metabolite levels by GC/MS

The metabolite contents presented in the current study were determined by a gas chromatography/mass spectrometry (GC/MS)-based qualitative and quantitative analysis of trimethylsilylated compounds. The tissue extraction, work-up and derivatization steps, as well as the details of the GC/MS parameters of this procedure have been described in detail previously (Hartmann et al., 2018; Yildiz et al., 2021). For the quantitative determination of metabolites, specific peaks of analytes and related internal standards from selected ion chromatograms were integrated [analyte (*m/z*) related to internal standard (*m/z*)]: Pip (*m/z* 156), related to D_9_-Pip (*m/z* 165); NHP (*m/z* 172), related to D_9_-NHP (*m/z* 181); SA (*m/z* 267), related to D_4_-SA (*m/z* 271); NHP-β-glucosid (NHPG) (*m/z* 172), NHP glucose ester (NHPGE) (*m/z* 172), SA-β-glucosid (SAG) (*m/z* 267) and SA glucose ester (SGE) (*m/z* 193): all related to salicin (*m/z* 268); camalexin (*m/z* 272), related to indole-3-propionic acid (*m/z* 202); Phe (*m/z* 218), Tyr (*m/z* 218), Trp (*m/z* 202), Val (*m/z* 144), Leu (*m/z* 158), Ile (*m/z* 158), α-aminoadipic acid (*m/z* 260): all related to norvaline (*m/z* 218); γ-tocopherol (*m/z* 488) and stigmasterol (*m/z* 484): both related to tocol (*m/z* 460). For absolute quantification of analytes, experimentally determined correction factors were considered. The metabolite levels were related to the FW of the leaf samples. Due to the unavailability of authentic compounds for NHPG and NHPGE, a relative quantification was performed (calculated numerical values are related to the sample FW and result from the consideration of correction factors with an assumed value of 1).

### Determination of transcript levels by RT-qPCR analysis

The transcript levels of specific genes were determined by RT-qPCR analysis using 50 mg of frozen and ground leaf tissue. The protocol of the RNA isolation, cDNA synthesis, and RT-qPCR steps has been previously outlined in detail (Návarová et al., 2012). As a reference gene, the *POLYPYRIMIDINE TRACT-BINDING PROTEIN 1* (*PTB1*) gene was used (Czechowski et al., 2005). The gene-specific primers used for RT-qPCR analysis are given in **Supplementary Table 1**. Expression value for each biological replicate was obtained by taking the mean of two technical replicates. Gene transcript levels were expressed relative to the mean value of the water-control sample.

### Statistical procedures

The numbers of biological replicates for each presented experiment are indicated in the figure legends. Numerical values of the bacterial and oomycete growth assays were log_10_-transformed and subject to ANOVA with *post-hoc* Tukey’s HSD test (significance level *P*<0.05 for each data subset; Hartmann et al., 2018). For metabolite and RT-qPCR-derived gene expression results, non-transformed numerical values were analysed by ANOVA with *post-hoc* Tukey’s HSD test (*P*<0.05) or by a non-parametric one-way ANOVA according to Kruskal-Wallis with stepwise step-down comparisons (*P*<0.05). The statistical analyses were performed with the SPSS^®^ statistical software (version 26; IBM^®^ Corporation). The depicted results were confirmed in at least one other independent experiment.

## Results

### NHP primes plants for enhanced flagellin-induced metabolic and transcriptional responses

Elevated levels of NHP in plants, either as a consequence of biological stimulation of its endogenous synthesis or because of exogenous treatment, trigger a primed state that boosts the *P. syringae*-induced accumulation of several (immune-related) metabolites and expression of defense-related genes (Návarová et al., 2012; Yildiz et al., 2021). To examine whether NHP would also prime a defined pattern stimulus, we comparatively investigated the metabolic response of Arabidopsis leaves to the peptide flg22 in naïve control plants and in NHP-pre-treated plants.

**Figure 1:**
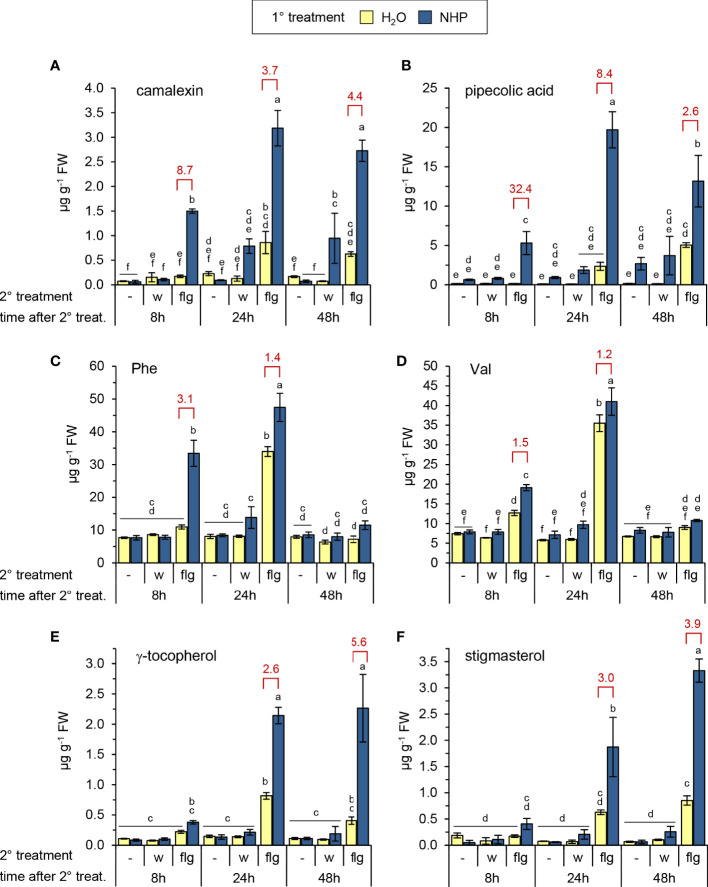
Exogenous N-hydroxypipecolic acid (NHP) primes Arabidopsis Col-0 plants for enhanced flg22-induced accumulation of (defense) metabolites. Single plants were watered with 10 ml of 1 mM NHP or 10 ml of H_2_O (1° treatment), and three leaves infiltrated one day later with a 1 µM aqueous solution of flg22-petide (flg) or water (w) as a mock-treatment (2° treatment). Metabolite levels of leaves were determined 8, 24, and 48 h after the 2° treatment. The leaves of a third set of plants were left untreated with respect to the 2° treatment (-), and leaf samples were harvested at the same time than those of the 2°-treated plants. One replicate sample consisted of six leaves from two plants. Accumulation of **(A)** camalexin, **(B)** pipecolic acid, **(C)** phenylalanine, **(D)** valine, **(E)** γ-tocopherol, and **(F)** stigmasterol [in µg g^-1^ fresh weight (FW)]. Bars represent means ± SD of three biological replicates (n = 3). Different letters denote significant differences (p < 0.05, ANOVA and *post-hoc* Tukey HSD test). The experiment was conducted twice with similar results. The degree of priming of flg22-responses is illustrated by a priming factor (red values). The factor is calculated by dividing the mean of the leaf metabolite levels of flg22-treated leaves from NHP-pretreated plants by the mean of those without pretreatment, if significant differences between these treatments were detected. See also **Supplementary Figures 1**–**3**.

**Figure 2:**
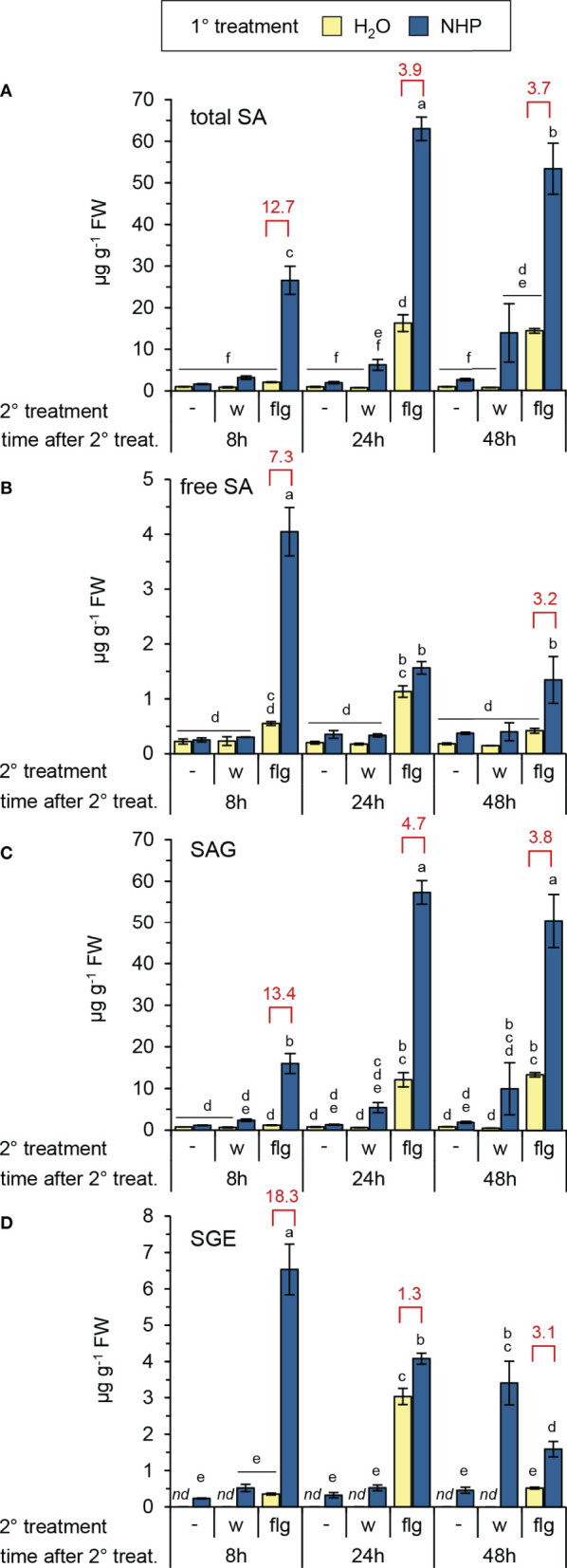
NHP primes Arabidopsis Col-0 for enhanced flg22-induced accumulation of salicylic acid and SA glucose conjugates. **(A)** total SA, i.e. the sum of free SA, SA-β-glucoside (SAG) and SA glucose ester (SGE), **(B)** free, unconjugated SA, **(C)** SAG, **(D)** SGE. Different letters denote significant differences (p < 0.05, ANOVA and *post-hoc* Tukey HSD test). Red values indicate the priming factor. For further details please refer to **Figure 1**.

Infiltration of leaves from naïve Arabidopsis Col-0 plants with a 1 µM solution of flg22 triggered the accumulation of the Trp-derived phytoalexin camalexin, the lysine-derived metabolites Pip, NHP and α-amino adipic acid, and the shikimate pathway-derived phenolic SA. In addition, increased levels of SA glucose conjugates (SA-β-glucoside [SAG] and SA glucose ester [SGE]) and the NHP N-O-glucoside NHPG were detected. Moreover, the flg22-treatment resulted in enhanced levels of the aromatic amino acids Phe, Tyr, and Trp, the branched-chain amino acids Val, Leu, and Ile, the vitamin E form γ-tocopherol, and the unsaturated phytosterol stigmasterol (**Figures 1**, **2**; **Supplementary Figures 1**, **2**). However, the flg22-treatment triggered a much weaker overall metabolic response than a bacterial challenge - with respect to both the quantities and the rates of compound accumulation (Griebel and Zeier, 2010; Návarová et al., 2012; Stahl et al., 2016; Hartmann et al., 2018; Stahl et al., 2019; Bauer et al., 2021; Yildiz et al., 2021). For example, while camalexin accumulated up to more than 100 µg g^-1^ fresh weight (FW) upon *Psm* attack at 48 h post inoculation (hpi) and was produced from about 10 hpi onwards in *Psm*-infected leaves (Stahl et al., 2016), it remained below 1 µg g^-1^ FW in the leaves of naïve, flg22-treated plants (**Figure 1**; **Supplementary Figure 3**). Moreover, accumulation of every of the examined metabolites was observed at 24 h but not yet at 8 h post flagellin treatment. Except for camalexin, Pip, SAG, NHPG, and stigmasterol, the flg22-induced metabolic increases in the naïve plants had transient character and declined at 48 h post treatment (**Figures 1**, **2**; **Supplementary Figures 1**–**3**).

Whereas a pre-treatment of plants with NHP had no direct effect on camalexin accumulation, it significantly accelerated and quantitatively enhanced the flg22-triggered biosynthesis of the phytoalexin (**Figure 1A**). In the leaves of NHP pre-treated plants, a marked accumulation of camalexin was already observed at 8 h after flg22-application, and this priming effect was discernible also at 24 and 48 h post flg22-treatment. To estimate the degree of NHP-induced priming, we calculated a priming factor (PF) as the ratio of the metabolite levels in flg22-treated leaves of NHP-pretreated plants and those in flg22-treated leaves of naïve plants at a given time-point (**Figures 1**, **2**; **Supplementary Figures 2**, **3**). For camalexin accumulation, the PFs amounted to 8.7, 3.7, and 4.4 for samples collected at 8 h, 24 h, and 48 h post flg22-treatment, respectively (**Figure 1A**). Priming of the flagelling-induced biosynthesis of camalexin was similarly observed when the NHP biosynthetic precursor Pip was exogenously applied to plants instead of NHP (**Supplementary Figure 3**).

NHP pre-treatment directly elevated Pip levels to a small extent but, more strikingly, resulted in an early and strong priming of the flg22-triggered generation of Pip (**Figure 1B**). At 8 h post flg22-treatment, naïve plants still contained basal levels of Pip, but NHP-pre-treated plants showed a significant flg22-induced Pip accumulation (PF = 32). This priming effect was still considerable at 24 h post flg22-treatment (PF = 8), with Pip accumulating to high levels in NHP-pre-supplied and flg22-treated plants (**Figure 1B**). Similarly, we observed an early and strong priming of the flg22-stimulated biosynthesis of SA, as indicated by priming factors of 13, 4, and 4 for the total levels of SA (per definition the sum of unconjugated SA, SAG, and SGE) at 8 h, 24 h, and 48 h post flg22-treatment, respectively (**Figure 2**). In this process, it was obvious that the flagellin-induced accumulation of SA and SGE were primed by NHP most strongly at 8 h post flg22-treatment (**Figures 2B, D**), while the priming of SAG occurred more steadily during the early and later phases of the experiment (**Figure 2C**).

The application of flg22 to the leaves of naïve Arabidopsis plants also significantly induced the accumulation of the amino acids Phe, Tyr, Trp, Val, Leu, and Ile at 24 h post treatment (**Figures 1C, D**; **Supplementary Figures 2A-D**). Following NHP pre-treatment of plants, flg22 triggered the accumulation of the three aromatic amino acids already at 8 h post treatment (PF 3.1, 2.3, and 2.7 for Phe, Tyr and Trp, respectively), while the NHP-induced priming was generally lower or even absent for the branched chain amino acids Val, Leu and Ile (PF always lower than 1.5). Further, flg22 induced the accumulation of γ-tocopherol and stigmasterol (**Figures 1E, F**), two non-polar metabolites whose production is stimulated by reactive oxygen species (ROS; Griebel and Zeier, 2010; Stahl et al., 2019). NHP pre-treatment significantly primed the production of γ-tocopherol and stigmasterol in later phases (24 and 48 h) after flg22-treatment but not yet at 8 h post application (**Figures 1E, F**). Similarly, NHP-mediated priming of the flg22-triggered accumulation of α-amino adipic acid, which is synthesized from Lys via the saccharopine pathway (Galili et al., 2001; Návarová et al., 2012), was observed in the later time-points after the application of the flg22-peptide (**Supplementary Figure 2E**).

**Figure 3:**
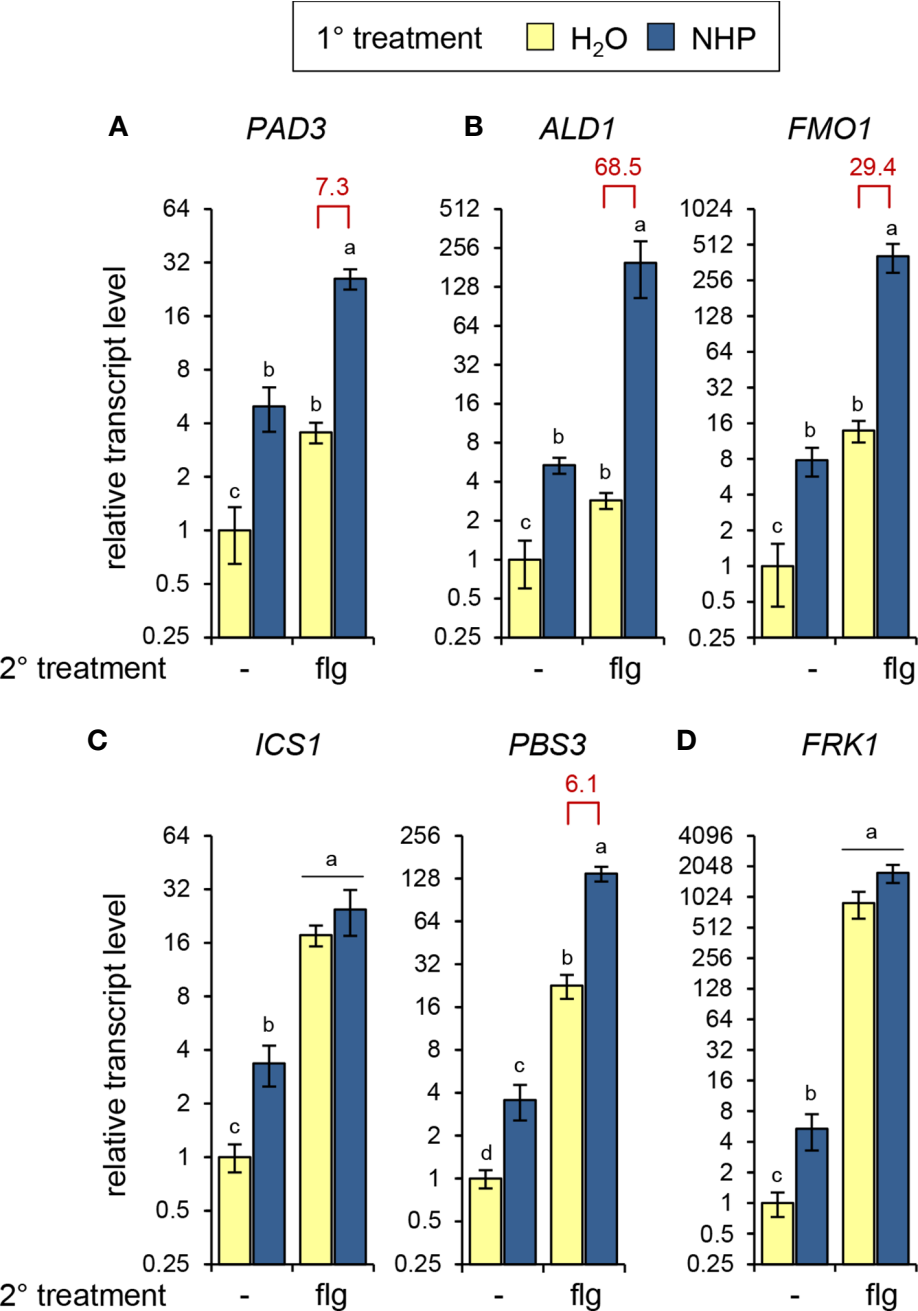
NHP primes Arabidopsis Col-0 plants for enhanced flg22-induced expression of genes involved in camalexin, Pip/NHP, and SA biosynthesis. Plants were 1°-treated with NHP or water, followed by a 2° treatment of the leaves with 1 µM flg22. Control plants were left untreated (-) with respect to the 2° treatment. Leaf samples, which consisted of 6 leaves from two plants, were harvested 8 h after the 2° treatment. Expression of **(A)** *PHYTOALEXIN-DEFICIENT3 (PAD3)* [camalexin biosynthesis], **(B)** *AGD2-LIKE DEFENSE RESPONSE PROTEIN1 (ALD1)* [Pip and NHP biosynthesis] and *FLAVIN-DEPENDENT MONOOXYGENASE1 (FMO1)* [NHP bionsynthesis], **(C)** *ISOCHORISMATE SYNTHASE1 (ICS1)* and *avrPphB SUSCEPTIBLE3 (PBS3)* [SA biosynthesis], and **(D)** *FLG22-INDUCED RECEPTOR-LIKE KINASE1 (FRK1)* was determined by RT-qPCR. Bars represent means ± SD of gene transcript levels calculated from three biological replicates (n = 3). The transcript levels for each gene are expressed relative to the mean value of the water-control sample. Different letters denote significant differences (p < 0.05, ANOVA and *post-hoc* Tukey HSD test). Red values indicate the priming factor. Further experimental details are described in **Figure 1**.

To examine whether the NHP-mediated priming of flagellin responses would be also apparent at the level of gene transcription, we assessed the flg22-induced expression of genes involved in the biosynthesis of camalexin [*PHYTOALEXIN-DEFICIENT3* (*PAD3*)], NHP (*ALD1* and *FMO1*), and SA (*ICS1* and *PBS3*), as well as expression of the strongly flagellin inducible gene *FLG22-INDUCED RECEPTOR-LIKE KINASE1* (*FRK1;* Asai et al., 2002) in the leaves of naïve and NHP-pretreated plants. Augmented NHP levels alone were sufficient to induce increased expression of any the genes under examination, and their transcript levels were elevated by factors between 3- and 8-fold following NHP treatment (**Figure 3**). Moreover, each gene exhibited significant responsiveness to flagellin. At 8 h post treatment, flg22 induced moderate elevations of *PAD3* and *ALD1* transcript levels (~ 3- to 4-fold), stronger increases of *FMO1*, *ICS1*, and *PBS3* transcript levels (~ 10- to 25-fold), and very strong (~ 900-fold) induction of *FRK1* expression (**Figure 3**). NHP-pre-treatment of plants markedly primed the leaves for the flg22-induced expression of *ALD1*, *FMO1*, *PAD3*, and *PBS3*, while the flg22-induced expression of *ICS1* and *FRK1* was hardly influenced (**Figure 3**). The significant priming of *PAD3* (PF = 7) and *ALD1* (PF = 69) expression corresponds to the priming of camalexin and Pip accumulation at the metabolic level, respectively (**Figures 1A, B**).

Together, our data indicate that NHP primes Arabidopsis plants for a stronger activation of flagellin-induced metabolic responses. Thereby, the degree and timing of priming might differ for different immune responses. This goes hand in hand with the observation that distinct flagellin-inducible genes can have different predisposition for an NHP-mediated, primed expression.

### NHP and SA mutually promote their biosynthesis in early stages of the compatible Arabidopsis-P. syringae interaction and additively enhance camalexin formation

**Figure 4:**
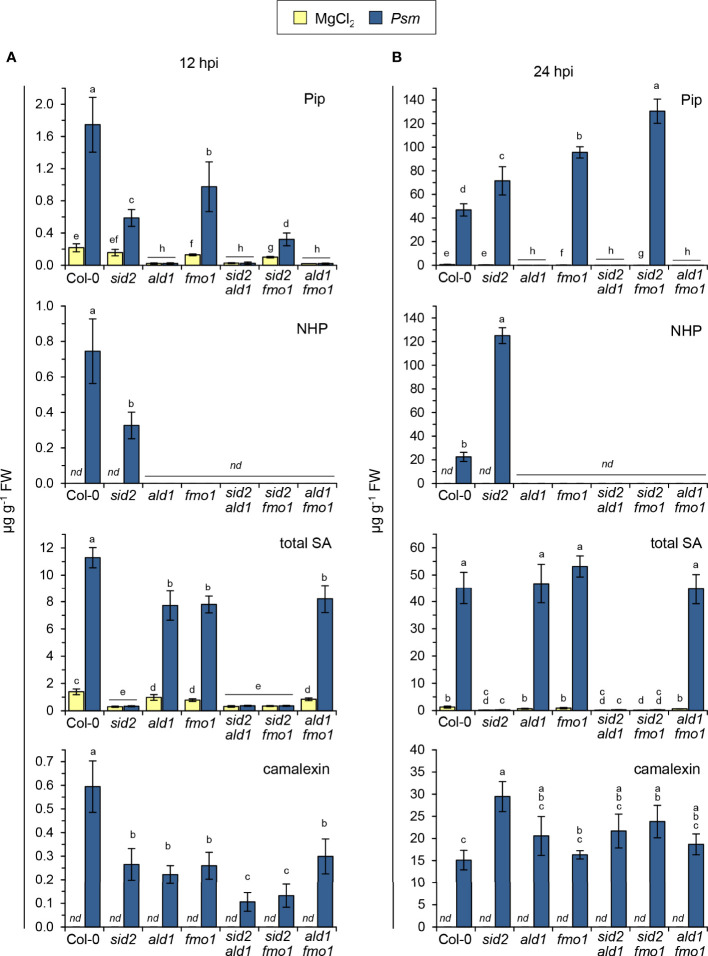
Accumulation of defense-related metabolites in Arabidopsis wild-type plants and mutant lines defective in NHP- and/or SA-biosynthesis at early [12 hours post inoculation (hpi)] and later (24 hpi) phases following bacterial inoculation. **(A)** Levels of Pip, NHP, and total SA (sum of SA, SAG and SGE) in leaves of Arabidopsis Col-0 (wild type), the single mutants *sid2-1, ald1*, and *fmo1* as well as the double mutants *sid2-1 ald1, sid2-1 fmo1*, and *ald1 fmo1* inoculated with compatible *P. syringae* pv. *maculicola* (*Psm*) at 12 hpi. Control plants were mock-treated with 10 mM MgCl_2_. **(B)** Accumulation of the same set of metabolites in leaves inoculated with *Psm* at 24 hpi. Bars represent means ± SD of five biological replicates (n = 5) for **(A)** and four biological replicates (n = 4) for **(B)**. One replicate sample consisted of six leaves from two plants. Different letters denote significant differences (p < 0.05, Kruskal-Wallis H test). *nd*: not detected. See also **Supplementary Figures 5**, **6**.

An interplay between accumulating NHP and SA is crucial for the establishment of biologically-triggered SAR in Arabidopsis (Bernsdorff et al., 2016; Chen et al., 2018; Hartmann et al., 2018; Yildiz et al., 2021). To further elucidate the interaction of the salicylate- and pipecolate pathways in mediating immune responses, we generated *sid2 ald1*, *sid2 fmo1*, and *ald1 fmo1* double mutants with the aim to compare their resistance characteristics with those of the respective single mutants and the Col-0 wild-type (**Figure 4**; **Supplementary Figure 4**; Bernsdorff et al., 2016). We first leaf-inoculated this set of Arabidopsis plants with the compatible *P. syringae* pv. *maculicoa* ES4326 (*Psm*) strain and then monitored accumulation of Pip, NHP, SA and their glycosylated derivates in the attacked leaves (**Figure 4**). As expected, single and double mutant plants lacking functional *ALD1* were unable to accumulate Pip, NHP as well as the NHP glucose conjugates NHPG and NHP glucose ester (NHPGE) upon *Psm* inoculation, while those possessing functional *ALD1* but lacking *FMO1* were able to generate Pip but not NHP and its derivates. Moreover, single and double mutants with *sid2* backgrounds were SA-induction deficient, failed to accumulate SA as well as its glucose conjugates SAG and SGE upon pathogen attack, and contained reduced basal SA levels (**Figure 4**; **Supplementary Figure 5**). Thus, direct comparisons of *sid2 ald1* or *sid2 fmo1* with the respective single mutants and the wild-type enabled us to study whether the execution of particular immune responses would require Pip, NHP and SA or a combination thereof. Further, comparison of defense phenotypes of Pip-accumulating *fmo1* with Pip-deficient *ald1 fmo1* and *ald1* allowed us to reassess whether an independent function of Pip beyond functioning as a precursor for immune-active NHP would exist. In addition, a comparison of the *ald1 fmo1* double mutant with the *ald1* single mutant was supposed to provide information about a hypothetical existence of an independent immune function of FMO1 beyond its role as Pip-N-hydroxylating NHP synthase.

At 12 h post inoculation with *Psm*, we observed that the accumulation of Pip and NHP in inoculated leaves was lower in *sid2* than in the Col-0 wild-type. Moreover, *sid2 fmo1* accumulated less Pip than *fmo1* (**Figure 4A**). This indicates that in this early interaction phase, SA favours the rises of the levels of the pipecolate pathway metabolites Pip and NHP. However, as observed previously for *Psm*-inoculated leaf samples harvested at 24 and 48 hpi (Hartmann et al., 2018; Yildiz et al., 2021), NHP over-accumulated in *sid2* at 24 hpi (**Figure 4B**). Therefore, in the leaves of naïve Arabidopsis plants inoculated with the compatible *Psm* strain, the regulatory impact of SA on the levels of NHP is double-edged: SA promotes NHP accumulation in the early interaction phase, while it acts as a negative modulator in the later stages of infection.

Further, we found that the Col-0 wild-type accumulated higher levels of total SA than *ald1*, *fmo1*, and *ald1 fmo1* at 12 h post *Psm* inoculation (**Figure 4A**). The attenuated biosynthesis of SA in the three mutant lines was most apparent when assessing the levels of SAG and SGE (**Supplementary Figure 5**). At 24 hpi, however, no differences between total SA levels in the wild-type and the NHP pathway mutants were detected (**Figure 4B**). Moreover, the accumulation of SA and its glucose derivates were always similar in *ald1*, *fmo1*, and *ald1 fmo1* (**Figure 4**; **Supplementary Figure 5**). Together, this indicates that NHP enhances the SA biosynthetic pathway at earlier biotic interaction phases, and that the NHP precursor Pip has no independent biological activity.

Interestingly, at 12 hpi, *fmo1* also accumulated Pip to lower levels than the wildtype (**Figure 4A**), suggesting that NHP is able to amplify the pathogen-induced production of its own biosynthetic precursor. As observed previously (Bernsdorff et al., 2016), *fmo1* over-accumulated Pip at 24 post *Psm* inoculation (**Figure 4B**), possibly because the inability of the mutant to further metabolize the at this stage more heavily accumulating Pip.

Previous experiments using Arabidopsis *sid* mutants suggested that the inducible accumulation of the phytoalexin camalexin in response to avirulent bacterial pathogens is negatively regulated by the SA pathway (Nawrath and Métraux, 1999). On one hand, our metabolite data confirmed this tendency because *Psm* inoculation resulted in a stronger camalexin accumulation in *sid2* at 24 hpi than in the wild-type or in the NHP-defective lines *ald1*, *fmo1* or *ald1 fmo1* (**Figure 4B**). On the other hand, the early production of camalexin at 12 h post *Psm* inoculation was attenuated in both the SA-deficient *sid2* plants and the NHP-deficient *ald1*, *fmo1* and *ald1 fmo1* lines. In addition, the SA- and NHP-deficient double mutants *sid2 ald1* and *sid2 fmo1* contained the lowest levels of camalexin at 12 hpi (**Figure 4A**). These results indicate that both SA and NHP promote the early biosynthesis of camalexin in the basal immune response of Arabidopsis to *Psm*, and that both immune signals additively contribute to the timely production of the phytoalexin.

### SA and NHP contribute additively or synergistically to Arabidopsis local resistance to pathogen infection

**Figure 5:**
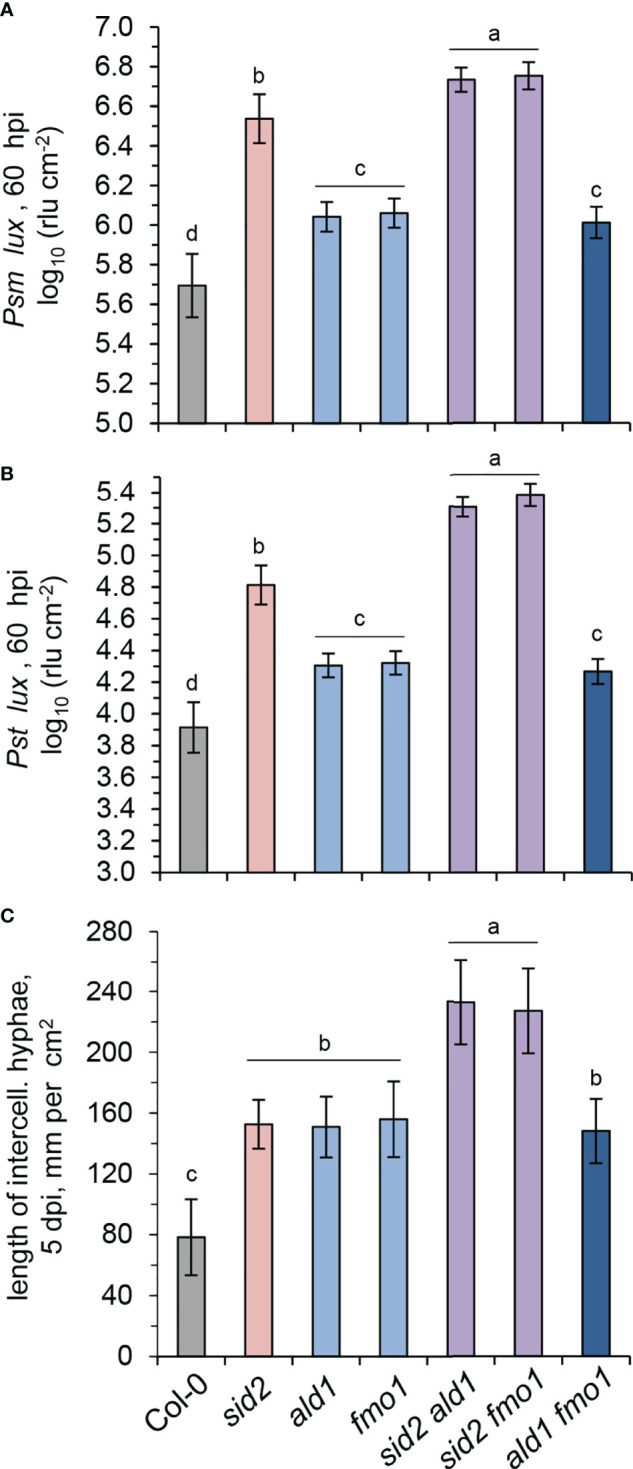
SA and NHP contribute additively to basal resistance of Arabidopsis to compatible bacterial and oomycete pathogens. **(A, B)** Basal resistance of Arabidopsis wild-type Col-0 and mutant lines defective in SA and/or NHP biosynthesis to virulent *Pseudomonas syringae* strains. **(A)** Naïve Arabidopsis plants of the indicated lines were inoculated with bioluminescent *P. syringae* pv. *maculicola* ES4326 *(Psm)* expressing the *luxCDABE* operon from *Photorhabdus luminescens* (*Psm lux*; Fan et al., 2008) by syringe-infiltration of three leaves each with a bacterial suspension of OD_600_ (optical density at 600 nm) = 0.001. As a measure of plant susceptibility, the numbers of bacteria were determined at 60 hpi in inoculated leaves by luminescence quantification and expressed as relative light units (rlu) per cm^2^ leaf area (Gruner et al., 2018). Bars indicate the mean ± SD of at least 15 leaf replicates (n ≥ 15). **(B)** Same experiment with *P. syringae* pv. *tomato* DC3000 *(Pst)* expressing *luxCDABE* (*Pst lux*; Fan et al., 2008) as the inoculating pathogen (OD_600 =_ 0.001; n ≥ 14). **(C)** Basal resistance of the indicated Arabidopsis lines to compatible *Hyaloperonospora arabidopsidis* isolate Noco2 (*Hpa*). The leaf rosette was spray-inoculated with a sporangial suspension of 2.5×10^4^ ml^-1^ and leaves harvested at 5 dpi. Invasively-growing intercellular hyphae (IH) within the leaf tissue were assessed at 5 days post inoculation (dpi) as a measure of disease severity and are given in mm IH per cm^2^ leaf area (Hartmann et al., 2018). The means (± SD) of ten leaves from different plants are given (n = 10). Different letters denote significant differences (p < 0.05, ANOVA and *post-hoc* Tukey HSD test).

To directly assess basal immunity to bacterial infection, we inoculated leaves of naïve Col-0, *sid2, ald1, fmo1, sid2 ald1, sid2 fmo1* and *ald1 fmo1* plants with the compatible *Psm* or *P. syringae* pv. *tomato* DC3000 (*Pst*) strains (**Figures 5A, B**). In both the *Psm*- and *Pst*-inoculation assays, bacterial growth was similar in *ald1, fmo1*, and *ald1 fmo1.* However, compared to the wild-type, the three NHP-deficient mutants showed increased susceptibility to both bacterial pathogens (**Figures 5A, B**). At the same time, the SA-induction-deficient *sid2* plants were more susceptible than the NHP-deficient pipecolate pathway mutants to *Psm* and *Pst* infection. Moreover, both *sid2 ald1* and *sid2 fmo1* were less resistant to both bacterial strains than *sid2* (**Figures 5A, B**).

Next, to specify the function of SA and NHP signalling in the basal immunity of Arabidopsis to oomycete infection, we inoculated the different lines under investigation with the oomycete pathogen *Hyaloperonospora arabidopsidis* isolate Noco2 (*Hpa*). *Hpa* is virulent to Arabidopsis Col-0 and able to establish invasive hyphal growth in the intercellular spaces of leaves (Slusarenko and Schlaich, 2003; Bartsch et al., 2006). We determined the lengths of intercellular hyphae in the leaves at 5 dpi as a measure of disease susceptibility of naïve plants (**Figure 5C**). Compared to the Col-0 wildtype, *ald1*, *fmo1*, and *sid2* single mutants showed a significantly stronger susceptibility to the oomycete, (**Figure 5C**). While the *ald1 fmo1* double mutant showed a similar susceptibility to *Hpa* than the *ald1* or *fmo1* single mutants, *sid2 fmo1* and *sid2 ald1* double displayed by far the strongest susceptibility of all of the lines under examination (**Figure 5C**).

Together, these resistance assays show that both NHP- and SA-initiated signalling contribute to basal immunity to *Psm*, *Pst* and *Hpa* infection, with a comparatively larger contribution of SA in the cases of bacterial attack. The similar basal immune phenotype of the NHP-deficient but Pip accumulating mutant *fmo1* and the *ald1* and *ald1 fmo1* lines that show both NHP- and Pip-deficiency confirm that NHP functions as the signal-active compound of the pipecolate pathway, and that Pip does not exhibit an independent immune-active function beyond its role as a necessary biosynthetic precursor for NHP (Hartmann et al., 2018; Zeier, 2021). The findings also emphasize that the immune function of FMO1 is restricted to its role in NHP formation in the pipecolate pathway. Finally, the high susceptibility of the *sid2 fmo1* and *sid2 ald1* double mutants indicate that the SA and NHP signalling pathways act additively to basal immunity against infection by compatible bacterial pathogens.

**Figure 6:**
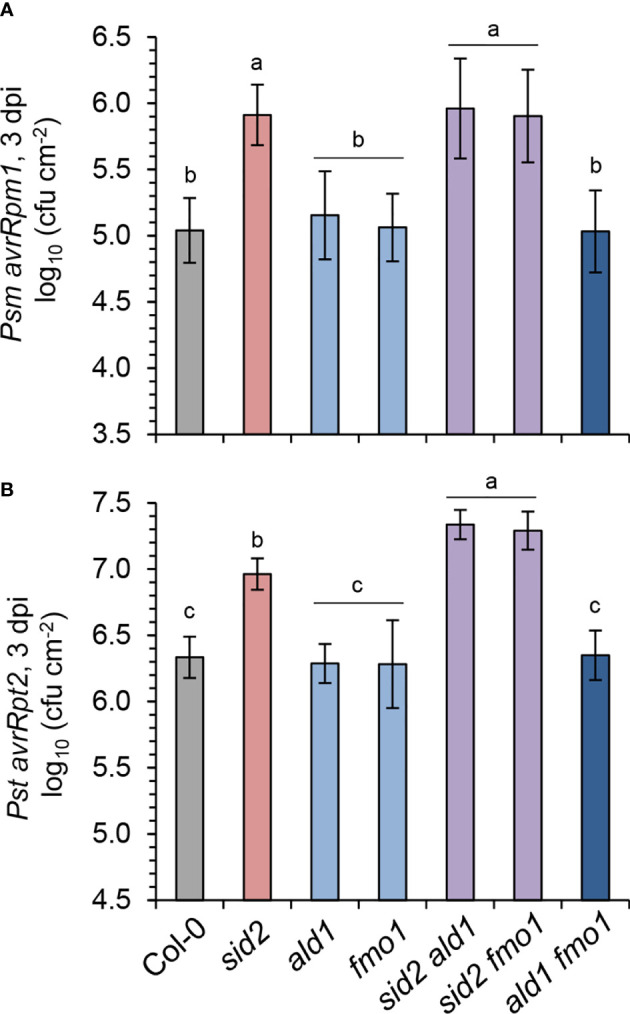
NHP fortifies the SA-mediated resistance to avirulent *P. syringae* triggered by the resistance protein RPS2. **(A)** Gene-for-gene resistance of the indicated Arabidopsis lines to avirulent *Psm avrRpm1*, which is recognized by the Rpm1 resistance protein. **(B)** Gene-for-gene resistance to avirulent *Pst avrRpt2* which is recognized by the Rps2 resistance protein. To assess plant resistance, three leaves of a plant were syringe-infiltrated as described in **Figure 5A** and bacterial numbers in leaves determined at 3 dpi by a plating-based assay. The means of colony-forming units (cfu) per cm^2^ leaf area ± SD of at least 9 replicate leaf samples (n ≥ 9) is given. Different letters denote significant differences (p < 0.05, ANOVA and *post-hoc* Tukey HSD test).

ETI is induced by the direct or indirect recognition of pathogen effector proteins by plant resistance proteins, which are commonly nucleotide binding/leucine-rich repeat (NLR)-type of immune receptors (Cui et al., 2015; Thordal-Christensen, 2020). For example, AvrRpm1 and AvrRpt2 are type III effectors from *P. syringae* whose cellular actions are recognized in Arabidopsis by the NLR receptors RESISTANCE TO P. SYRINGAE PV MACULICOLA1 (RPM1) and RESISTANCE TO P. SYRINGAE2 (RPS2), respectively (Mackey et al., 2002; Axtell and Staskawicz, 2003). To compare the contributions of the SA and NHP pathways in these distinct ETI responses, we inoculated leaves of the lines under investigation with *Psm* expressing AvrRpm1 (*Psm avrRpm1*) or *Pst* expressing AvrRpt2 (*Pst avrRpt2*), and scored bacterial growth at 3 dpi (**Figure 6**). Upon *Psm avrRpm1* inoculation, all the lines harboring mutations in *sid2* were more susceptible than the wild-type or lines with defects in the NHP pathway, indicating that the SA pathway is required but the NHP pathway is dispensable for local, RPM1-mediated resistance (**Figure 6A**). The growth of *Pst avrRpt2* was also similar in Col-0 and the pipecolate pathway mutants *ald1*, *fmo1* and *ald1 fmo1*, while *sid2* again showed an increased susceptibility (**Figure 6B**). This emphasizes a particular importance of the SA pathway for RPS2-mediated ETI, while NHP seemed to be dispensable in this case. However, the resistance to *Pst avrRpt2* was lower in the *sid2 ald1* and *sid2 fmo1* double mutants than in the *sid2* single mutant. Thus, the *Pst avrRpt2* -related growth data indicate that NHP contributes to RPS2-mediated ETI in the absence of a functional SA pathway, while intact SA biosynthesis masks this contribution in the pipecolate pathway mutants (**Figure 6B**).

### The flagellin-triggered acquired resistance response in local tissue shows mechanistic similarities and differences to SAR

**Figure 7:**
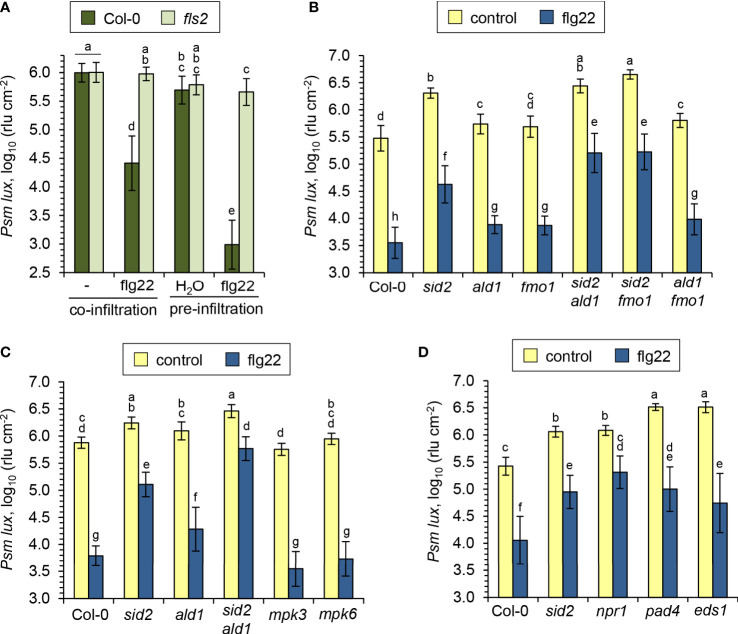
SA and NHP additively contribute to the flagellin-induced acquired resistance response in Arabidopsis leaves. **(A)** Comparison of resistance induction by the flagellin peptide flg22 on leaves of Arabidopsis Col-0 and mutants defective in the flagellin receptor *FLS2*. Flg22 and compatible *Psm lux* were either co-applied to leaves, or flg22 was applied prior to bacteria inoculation. Pre-application: Three leaves per plant were syringe-infiltrated with an aqueous solution of 1 µM flg22 as an inducing treatment or with water as a control treatment. One day later, the same leaves were syringe-inoculated with *Psm lux* and bacterial numbers assessed 60 h later as described in **Figure 5A**. Co-application: Bacterial suspensions of *Psm lux* (OD_600 =_ 0.001) containing (flg22) or lacking (-) 1 µM of flg22 were infiltrated into leaves and bacterial numbers scored 60 h later. Bars show the mean ± SD of the rlu values of at least 15 leaf replicates (n ≥ 15). **(B-D)** Flg22-induced resistance in the leaves of Arabidopsis wild-type Col-0 and different defense mutant lines, as assessed by the co-application procedure. Bars show the mean ± SD of 15 **(B, C)** or 18 **(D)** replicate leaf samples. Different letters denote significant differences (P < 0.05, ANOVA and *post-hoc* Tukey HSD test).

A pre-treatment of plants with bacterial flagellin induces a strong acquired resistance response to subsequent infection by virulent pathogens in the treated tissue, (Zipfel et al., 2004; Tsuda et al., 2009), and we now aimed at specifying the role of the NHP pathway in this context. To test for flagellin-induced acquired resistance, we first suspended the *Psm* bacteria either in 10 mM MgCl_2_ containing 1 µM flg22 or in a 10 mM MgCl_2_ control solution, inoculated Arabidopsis leaves, and compared bacterial numbers at 2.5 dpi for both treatments. The co-application with flg22 in this assay resulted in a strong reduction of bacterial growth in the Col-0 wildtype compared to the control condition, and this resistance effect was entirely absent in a flagellin-insensitive *fls2* mutant (**Figure 7A**). In a variation of this assay, we pre-infiltrated the leaves of Arabidopsis plants with an aqueous solution of 1 µM flg22 or with water, challenged the same leaves one day later with *Psm lux*, and scored bacterial numbers another 2.5 days later. In these assays, we observed an even larger resistance induction in the Col-0 wildtype. Again, the flagellin-induced resistance was fully depended on a functional *FLS2* gene (**Figure 7A**). However, when comparing pre- with co-infiltration, a modest resistance-enhancing effect of the pre-infiltration procedure alone was apparent (**Figure 7A**). We therefore decided to use the co-infiltration assay for further experiments with NHP-, SA-, and other immune-related pathway mutants to test for the flagellin-induced resistance response.

**Figure 8:**
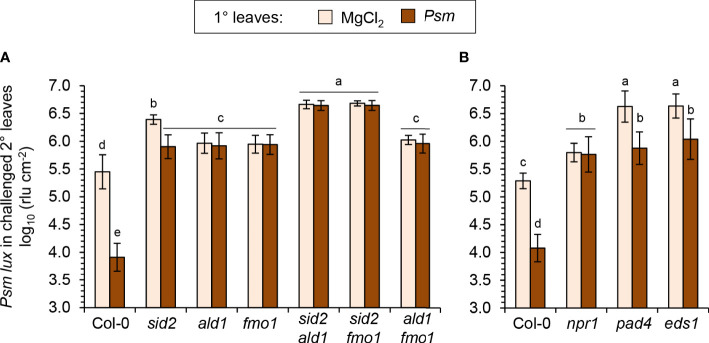
Establishment of systemic acquired resistance (SAR) triggered systemically by bacterial inoculation and the locally assessed, flg22-triggered acquired resistance response are based on both overlapping and distinct signaling principles. **(A, B)** To assess SAR in Arabidopsis, three lower (1°) leaves per plant were either inoculated with *Psm* (OD_600_ = 0.005) or mock-infiltrated with 10 mM MgCl_2_. Two days after this 1°-inducing treatment, three upper (2°) leaves were challenge-inoculated with *Psm lux* (OD_600_ = 0.001), and bacterial numbers in the 2° leaves scored 2.5 days after the challenge-inoculation (see **Figure 5A** for details). **(A)** SAR assay with Col-0 wildtype, NHP- and SA-pathway single and double mutants. **(B)** SAR assay with Col-0, *npr1-3*, *pad4-1*, and *eds1-2* mutant plants. Bars show the mean ± SD of at least 15 leaf replicates (n ≥ 15). Different letters denote significant differences (P < 0.05, ANOVA and *post-hoc* Tukey HSD test).

The NHP pathway mutants *ald1*, *fmo1* and *ald1 fmo1* showed a lower degree of resistance induction by flg22-treatment than Col-0 plants, indicating a contribution of NHP to the locally-induced flagellin response (**Figures 7B, C**). Still, however, a similarly pronounced and considerable resistance induction was observed in these three lines, demonstrating that parallel signalling pathways act at least in part independently from NHP to mediate flagellin-induced acquired resistance. This is in sharp contrast to the systemic, pathogen-inducible SAR response, for which NHP is indispensable. This is underlined by the full incompetency of the NHP pathway mutants *ald1*, *fmo1* and *ald1 fmo1* to induce any measurable SAR effect (**Figure 8A**; Song et al., 2004; Mishina and Zeier, 2006; Návarová et al., 2012; Bernsdorff et al., 2016). Previous results have revealed a marked contribution of the SA pathway in flagellin-induced resistance (Zipfel et al., 2004; Tsuda et al., 2009), which was confirmed in our analyses by an attenuated response to flg22 in the SA induction deficient *sid2* line (**Figures 7B-D**). The attenuation of flagellin-induced resistance was more pronounced in *sid2* than in *ald1*, *fmo1* or *ald1 fmo1*, indicating a stronger contribution of SA than of NHP to this response (**Figures 7B, C** **)**.

Although *sid2* shows a strongly diminished establishment of SAR, it has the competency of a weak pathogen-inducible SAR that is not detected in *sid2 ald1* or *sid2 fmo1* (**Figure 8A**). This corroborates our previous finding that the NHP-triggered SAR response is strongly amplified by but does not entirely depend on SA (Bernsdorff et al., 2016; Yildiz et al., 2021). Importantly, the *sid2 ald1* and *sid2 fmo1* double mutants showed a weaker flagellin-induced resistance than both *sid2* on one hand, and *ald1*, *fmo1* or *ald1 fmo1* on the other hand (**Figures 7B, C** **)**. Therefore, additive contributions of the SA and NHP pathways also exist for the establishment of flagellin-triggered acquired resistance. However, although markedly attenuated, a significant flg22-response was even detected in *sid2 ald1* and *sid2 fmo1*, indicating that immune signals other than SA and NHP independently contribute to flagellin-induced acquired resistance (**Figures 7B, C**).

Since flg22-treatment induces MAPK cascades, in particular the activation of MPK3 and MPK6 (Tsuda et al., 2009; Frei dit Frey et al., 2014), we tested flagellin-induced resistance in *mpk3* and *mpk6* knockout mutants. However, these lines showed a wildtype-like resistance induction in response to flg22-treatment. Moreover, we tested mutant lines with defects in genes coding for the immune-regulatory proteins PHYTOALEXIN-DEFICIENT4 (PAD4), ENHANCED DISEASE SUSCEPTIBILIY1 (EDS1) and NPR1 for their abilities to induce flg22-triggered resistance and SAR (Feys et al., 2001; Dong, 2004; Bartsch et al., 2006). The *pad4* and *eds1* mutants, which were highly susceptible to *Psm* in the naïve, uninduced state (**Figures 7D**; **8B**), showed a considerable flg22-response and increased resistance to similar levels than flagellin-treated *sid2* but to lower levels than the flagellin-induced wild-type (**Figure 7D**). Moreover, both *pad4* and *eds1* plants were able to establish a diminished but still significant *Psm*-triggered SAR (**Figure 8B**). The *npr1* mutant, by contrast, which is largely insensitive to both SA- and NHP-inducible immunity (Delaney et al., 1995; Liu et al., 2020; Yildiz et al., 2021), showed a fully compromised SAR and exhibited a weaker flg22-response than the SA-deficient *sid2* line (**Figures 7D**; **8B**; Yildiz et al., 2021).

Together, these mutant analyses show that SA and NHP additively contribute to the local immune response triggered by flg22-treatment but that other defense signalling pathways exist that provide independent, additional contributions. SAR, by contrast, does not develop in the absence of NHP biosynthesis and also largely dependents on the ability of plants to accumulate SA. This reveals both overlapping principles and differences for the signalling mechanisms that culminate in local acquired resistance induced by exogenous flagellin and the systemic SAR response.

## Discussion

### N-Hydroxypipecolic acid boosts diverse flagellin-induced metabolic and transcriptional responses to different degrees

Plants exhibiting SAR are primed to systemically defend themselves more quickly and vigorously against subsequent pathogen attack. A series of recent findings indicate that NHP functions as a key mediator of SAR-associated priming to bacterial infection (Zeier, 2021). In the current study, we investigated whether NHP would also amplify metabolic and transcriptional responses of Arabidopsis to bacterial flagellin as a defined molecular pattern. This allowed to compare priming of plant responses elicited by the single, quantitatively constant stimulus flagellin with the priming of responses associated with the more complex plant-bacterial interaction (Návarová et al., 2012; Bernsdorff et al., 2016; Hartmann et al., 2018; Yildiz et al., 2021).

**Figure 9:**
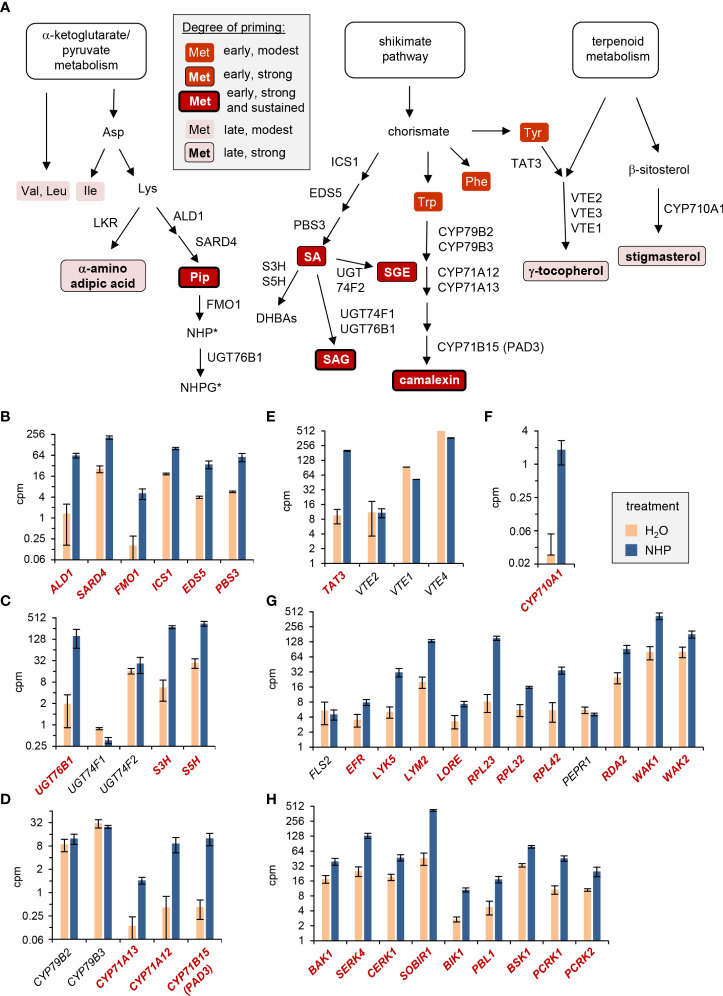
NHP-triggered priming of flagellin-inducible metabolic responses and direct induction of selected immune-related genes by NHP. **(A)** NHP primes the flagellin-induced induction of defense-related metabolic pathways to different degrees. The temporal sequence and magnitude of the distinct priming effects are indicated by different background colors, framings and font-weights of the descriptors of metabolites, as indicated in the grey-shaded legend. Abbreviations of the enzymes catalyzing individual reaction steps are indicated next to the arrows. Abbreviations not outlined in the main text: EDS5, ENHANCED DISEASE SUSCEPTIBILITY5; DHBAs, dihydroxybenzoic acids; S3H, salicylate-3-hydroxylase; S5H, salicylate-5-hydroxylase; LKR, lysine-ketoglutarate reductase; UGT, Uridine-diphosphate-dependent glycosyltransferase; CYP, cytochrome P450 monooxygenase; VTE, VITAMIN E DEFICIENT. *: The experimental design did not allow a direct assessment of the priming of the accumulation of NHP and its derivates at the metabolic level (see discussion). **(B-H)** Direct transcriptional response of Arabidopsis Col-0 plants to NHP. The depicted bar graphs display the means of expression levels (counts per million, cpm) of genes in the leaves of NHP-treated (blue) or H_2_O-treated (light red) control plants, as assessed by RNA-sequencing-based analyses (Yildiz et al., 2023). For the genes displayed in bold and red, significant differences (false discovery rate [FDR] < 0.05) between the NHP- and control-treatments exist. **(B-F)** Genes involved in defense-related metabolic pathways: **(B)** biosynthesis of SA and NHP, **(C)** NHP and SA metabolism, **(D)** camalexin biosynthesis, **(E)** vitamin E biosynthesis, and **(F)** stigmasterol biosynthesis. **(G, H)** Genes involved in pattern perception and early signaling: **(G)** pattern recognition receptors, and **(H)** co-receptors and receptor-like cytoplasmic kinases. Abbreviations not outlined in the main text: LYK5, LYSM-CONTAINING RECEPTOR-LIKE KINASE 5; LYM2, LYSM DOMAIN GPI-ANCHORED PROTEIN 2; LORE,LIPOOLIGOSACCHARIDE-SPECIFIC REDUCED ELICITATION; RPL, receptor-like protein; PEPR1, PEP1 RECEPTOR1; RDA2, RESISTANT TO DFPM INHIBITION OF ABA SIGNALING 2; WAK1/2, CELL WALL-ASSOCIATED KINASE1/2; SOBIR1, SUPPRESSOR OF BIR1 1; BSK1, BRASSINOSTEROID-SIGNALING KINASE1; PCRK1/2, PTI COMPROMISED RECEPTOR-LIKE CYTOPLASMIC KINASE1/2.

Our findings show that the NHP-triggered priming of metabolic reactions in Arabidopsis leaves associated with bacterial challenge and flagellin exposure are qualitatively and quantitatively very similar (**Figure 9A**; Yildiz et al., 2021). NHP induced early, strong and sustained priming of the flg22-induced accumulation of camalexin and Pip, and of the flg22-induced activation of the SA biosynthetic pathway (**Figures 1**; **2**; **9A**). More specifically, while the flg22-induced accumulation of SA and SGE was primed most strongly in the early phases after flg22-treatment, a strong priming of SAG production occurred continuously, also in later stages after flagellin exposure (**Figure 2**). This illustrates that the metabolic flow of the SA pathway is finally directed to SAG as a dominant storage form (Klessig et al., 2018). Priming of the pathogen-inducible, terpenoid pathway-derived and non-polar metabolites γ-tocopherol and stigmasterol was also strong but occurred mainly at later times following flg22-treatment (**Figures 1E, F**). An early but more modest priming was observed for the accumulation of the aromatic amino acids Phe, Tyr, and Trp (**Figure 1C**; **Supplementary Figure 2**). Notably, Trp and Tyr function as metabolic precursor for the biosynthesis of the priming-affected metabolites camalexin and γ-tocopherol, respectively (**Figure 9A**). And finally, a weak priming of the accumulation of the branched chain amino acids Val, Leu and Ile, which already markedly accumulated upon flg22-exposure alone, was observed at later times after elicitor treatment (**Figures 1D**, **9A**; **Supplementary Figure 2**). Therefore, NHP primes the flg22-induced generation of a large portion of previously described metabolites that accumulate upon infection with compatible *Psm* bacteria in Arabidopsis leaves (Griebel and Zeier, 2010; Návarová et al., 2012; Stahl et al., 2016; Stahl et al., 2019; Yildiz et al., 2021). However, the degree and temporal sequence of priming vary between these distinct metabolic pathways (**Figure 9A**).

How does NHP prime plants for an enhanced defense capacity? Recent RNA-sequencing-based analyses show that exogenous NHP induces a direct transcriptional response in Arabidopsis leaves that includes up-regulation of about 3000 genes (Yildiz et al., 2021; Yildiz et al., 2023). This response is largely similar to the transcriptional reprogramming that occurs during biological SAR in the distant leaf tissue of locally pathogen-inoculated plants (Bernsdorff et al., 2016). Similarly, exogenous NHP triggered a significant transcriptional response in wheat seedling that up-regulated a battery of SAR-related genes (Zhang et al., 2021). Notably, the direct transcriptional response to NHP in Arabidopsis includes up-regulation of key biosynthetic genes of the primed metabolic pathways (Yildiz et al., 2021; Yildiz et al., 2023). For example, NHP up-regulates all of the genes required for the stress-inducible biosynthesis of NHP (*ALD1, SARD4, FMO1*), SA (*ICS1, EDS5, PBS3*), as well as key genes of SA and NHP glucosylation (*UGT76B1*) and SA hydroxylation (**Figures 9B, C**; Zeier, 2021). This is consistent with the strong NHP-mediated priming of flg22-triggered accumulation of Pip, SA and SA glucose conjugates (**Figures 1**; **2**). Since we fed plants with exogenous NHP solution in the priming assays, it was not possible in our metabolite analyses to discriminate between endogenously accumulating NHP and exogenously administered and subsequently absorbed NHP, which prevents direct information about the priming of NHP and its glucose derivates at the metabolic level. However, the priming of Pip in parallel with the direct up-regulation and primed flg22-triggered expression of *FMO1* strongly suggests that NHP is able to fortify its own biosynthesis (**Figure 3**). NHP also enhances transcription of the three cytochrome P450 monooxygenase genes *CYP71A12, CYP71A13* and *CYP71B15/PAD3* that are involved in the biosynthesis of camalexin. By contrast, *CYP79B2* and *CYP79B3* that encode the enzymes catalysing the entrance reaction into this Trp catabolic pathway to camalexin show strong constitutive expression but are not up-regulated by NHP (**Figure 9D**). This suggests that the NHP-triggered transcriptional response generally elevates the enzymatic equipment to generate defense-related metabolites. In particular, NHP tends to augment the transcript levels of biosynthetic genes with lower constitutive, basal expression, which might help to fill in enzymatic gaps of a particular pathway. Consistently, the priming of γ-tocopherol and stigmasterol accumulation were associated with an NHP-induced up-regulation of the stress-inducible pathway genes *TYROSINE AMINOTRANSFERASE3* (*TAT3*) and *CYP710A1*, respectively (**Figures 9E, F**). While TAT3 supposedly acts relatively early in the tocopherol biosynthetic pathway, CYP710A1 catalyses the final step in the biosynthesis of stigmasterol (**Figure 9A**), indicating that both earlier and later pathway genes might act as switches for the priming of stress-inducible metabolic pathways.

Besides directly promoting the biosynthetic pathways of stress-inducible metabolites, analyses of the transcriptional SAR and NHP responses also indicate that NHP enhances the responsiveness of plant cells at the levels of pathogen perception and associated downstream signalling (Bernsdorff et al., 2016; Hartmann et al., 2018; Yildiz et al., 2021). The major part of plant surface receptors that monitor specific extracellular molecular cues to activate intracellular output programs constitute receptor kinases (RKLs) and receptor-like proteins (RLPs), the latter in combination with interacting adaptor kinases (Gust and Felix, 2014). Among both RLKs and RLPs, a series of PTI-related pattern recognition receptors (PRRs) have been identified (**Figure 9G**; DeFalco and Zipfel, 2021). Upon binding of peptide- or small molecule-ligands, these surface receptor units associate with co-receptors of the SOMATIC EMBRYOGENESIS RECEPTOR KINASE (SERK)-type family to initiate transphosphorylation and further downstream signalling events (Ma et al., 2016). Moreover, receptor-like cytoplasmic kinases (RLCKs) combine with these surface receptor complexes for intracellular signal transduction (Liang and Zhou, 2018).

Upon binding of its ligand flagellin, the RLK FLS2 interacts with its co-receptor, BAK1 (BRI1-associated receptor kinase1), to form an active pattern recognition receptor complex that triggers flagellin responses (Chinchilla et al., 2007; Heese et al., 2007). In addition, the RLCK BOTRYTIS-INDUCED KINASE1 (BIK1) and its closest homologue PBS1-LIKE1 (PBL1) combine with FLS2 to mediate flagellin-induced signal transduction (Lu et al., 2010; Ranf et al., 2014; Liang and Zhou, 2018). Whereas *FLS2* transcription is not regulated by NHP, expression of the co-receptor gene *BAK1* and the RLCK genes *BIK1*, *PBL1, BSK1, PCRK1, and PCRK2*, whose gene products are involved in FLS2-mediated signal transduction (Liang and Zhou, 2018), is significantly enhanced by NHP (**Figures 9G, H**). Therefore, NHP up-regulates several components of the flagellin-sensing receptor complex. An increased number of functional receptor units might more effectively perceive flagellin molecules and contribute to the observed NHP-mediated priming of flg22-responses. A similar scenario is likely to occur for the perception of other bacterial PAMPs, fungal PAMPs and DAMPs, since the transcripts of genes coding for the elongation factor-Tu receptor EFR (Zipfel et al., 2006), the lipopolysaccharide receptor LORE (Ranf et al., 2015), the chitin-sensing receptor kinases CERK1, LYK5 and LYM2 (Miya et al., 2007; Faulkner et al., 2013; Cao et al., 2014), and other characterized PRRs show significant NHP-triggered accumulation. These also include a series of RLPs and the adaptor kinase SOBIR1 (**Figures 9G, H**; Liebrand et al., 2013; Zhang et al., 2014; Albert et al., 2015; Fan et al., 2022). Therefore, NHP most likely primes plants for enhanced immune responses to other microbial patterns as well, which remains, however, to be experimentally verified.

Besides inducing a direct transcriptional response, our data indicate that NHP also primes the flg22-triggered expression of defense-related genes (**Figure 3**). We observed a boosted activation of biosynthetic genes of the camalexin (*PAD3*), the pipecolate (*ALD1, FMO1*), and the SA (*PBS3*) pathways in this context (**Figure 3**). The priming at the levels of biosynthetic gene expression might explain why the NHP-mediated enhancement of the flg22-induced accumulation of camalexin, Pip and SA pathway products is particularly strong (**Figure 9A**). Interestingly, we found that some flagellin-inducible genes show a weak predisposition for NHP-mediated priming. For example, *FRK1*, a classical marker gene for flagellin responses (Asai et al., 2002), and *ICS1* showed strong flg22-induced expression that was hardly affected by NHP pre-treatment. Whether genes with very strong stimulus-induced expression do generally exhibit a weaker predisposition for (NHP-mediated) priming than such with lower stimulus-induced expression is an interesting hypothesis that, however, cannot be generalized from these few examples.

The strong priming of the flagellin-induced biosynthesis of the Arabidopsis phytoalexin camalexin by NHP is consistent with the heavily primed accumulation of camalexin in response to *P. syringae* challenge in NHP- and SAR-induced plants (Návarová et al., 2012; Bernsdorff et al., 2016; Yildiz et al., 2021). A reduced NHP-triggered camalexin priming was observed in the SA-induction-deficient *sid2* mutant, indicating that SA amplifies the priming program induced by NHP (Yildiz et al., 2021). Interestingly, priming of *P. syringae-* and *Botrytis cinerea*-induced camalexin accumulation was also observed in plants exhibiting induced systemic resistance (ISR) (Nguyen et al., 2022). ISR is induced by beneficial bacteria in the root and mechanistically different to SAR. Nevertheless, analyses of SA pathway mutants suggest that SA fortifies also the ISR-triggered priming of camalexin accumulation (Nguyen et al., 2022). Whether NHP is also involved in the ISR-associated priming process remains to be determined. A recent study indicates that, in addition to immune-active metabolites, epigenetic modifications such as histone methylation and acetylation or DNA demethylation are involved in mediating the speed of camalexin biosynthesis (Zhao et al., 2021; Huang et al., 2022). The boosted biosynthesis of phytoalexins and other antimicrobial secondary metabolites is commonly observed among plants in inherently distinct metabolic pathways. Notably, besides priming of the biosynthesis of the Trp-catabolite camalexin in Arabidopsis (Návarová et al., 2012), activation of the pipecolate pathway by exogenous Pip treatment in tobacco triggers priming of *P. syringae*-induced accumulation of the Orn-derived pyrrolidine alkaloid nicotine (Vogel-Adghough et al., 2013). Moreover, the synthetic priming inductors S-acibenzolar-S-methyl or dichloroisonicotinic acid primed the pathogen-induced accumulation of isoflavonoid phytoalexins in cowpea (Latunde-Dada and Lucas, 2001), the elicitor-triggered secretion of coumarins in parsley cells (Kauss et al., 1992), and the expression of biosynthetic genes of diterpenoid phytoalexins in rice (Akagi et al., 2014), just to name a few examples.

In this study, we have focussed on the assessment of flagellin-triggered metabolic and transcriptional responses and demonstrated a significant role of NHP in conditioning these responses. Well-characterized cell wall-based defenses following flagellin perception are the deposition of callose into cell walls and ROS accumulation (Asai et al., 2002). Interestingly, exogenous treatment with NHP was shown to elevate expression of cell wall fortification enzymes and callose deposits in wheat seedlings (Zhang et al., 2021), suggesting that NHP might prime PTI-related cell wall-based defense reactions as well. In Arabidopsis, SA and jasmonate signaling enhanced both the flg22-triggered callose deposition and oxidative burst (Yi et al., 2014). These findings indicate a relevance for hormone-based priming mechanisms of flagellin-triggered cell wall-based immunity.

### SA and NHP provide synergistic and additive contributions to PTI- and ETI-related local immunity and early camalexin accumulation in non-primed plants

Using a complete set of metabolically well-characterized Arabidopsis lines impaired in the pipecolate and/or SA biosynthetic pathways (**Figure 4**; **Supplementary Figures 4**–**6**), we also revisited the role of the NHP pathway as well as the interplay of NHP and SA in local immune responses in naïve, non-primed plants (**Figures 4**–**6**). In the compatible interaction between *Psm* and Arabidopsis, Pip and NHP usually start to accumulate in inoculated leaves from 10 hours post inoculation onwards, while SA biosynthesis is induced some hours earlier (**Figure 4**; **Supplementary Figure 5**; Hartmann et al., 2018; Hartmann and Zeier, 2019). Comparative metabolite analyses in the early *Psm* – Arabidopsis interaction phase (i.e., at 12 hpi) showed that a failure of NHP accumulation (e.g., in *ald1*, *fmo1* and *ald1 fmo1*) results in an attenuated biosynthesis of SA, while a lack of SA accumulation (in *sid2*) negatively affects Pip and NHP production (**Figure 4**; **Supplementary Figure 5**). Thus, the rising levels of NHP in the early compatible plant-bacterial interaction intensify SA production, while at the same time, accumulating SA positively influences Pip and NHP biosynthesis (**Figure 4A**). Interestingly, the local, *Psm*-induced expression of *FMO1* was over-proportionally attenuated at 10 hpi in the *sid2 ald1* double mutant compared to either of the single mutants, corroborating the here-described synergistic interplay of NHP and SA in the induction of early basal defense responses (Bernsdorff et al., 2016).

Previous work indicated a positive influence of functional *ALD1* on the *P. syringae*-triggered accumulation of camalexin, while *SID1* and *SID2* exhibited negative impact on its accumulation (Nawrath and Métraux, 1999; Song et al., 2004). Our results suggest positive influences of both the NHP and SA pathways on the biosynthesis of camalexin in the early *Psm*-Arabidopsis interaction, because both *ald1*, *fmo1*, *ald1 fmo1* and *sid2* showed lower camalexin accumulation than the Col-0 wild-type at 12 hpi (**Figure 1A**). Moreover, a direct comparison of *sid2 ald1* or *sid2 fmo1*, which are both SA- and NHP-deficient, with the respective single mutants and the wild-type show that the early generation of camalexin is additively promoted by SA and NHP and occurs most efficiently when both immune signals are present (**Figure 4A**). Therefore, a positive interplay between NHP and SA, that was primarily described in context with SAR-induced, primed plants in previous studies (Bernsdorff et al., 2016; Hartmann et al., 2018; Zeier, 2021), also exists in early basal resistance responses of naïve, unprepared plants at sites of pathogen inoculation.

At later phases of the local *Psm*-Arabidopsis interaction (e.g., 24 to 48 hpi), a positive impact of NHP signalling on SA biosynthesis or of SA signalling on NHP biosynthesis is not apparent, because at these times, Pip and NHP accumulate to at least wild-type levels in the SA-induction-deficient *sid2* plants, and because NHP-deficient *ald1*, *fmo1* or *ald1 fmo1* plants showed no defect in SA accumulation (**Figure 4B**; Návarová et al., 2012; Bernsdorff et al., 2016). In fact, NHP and its glucose ester NHPGE even over-accumulate in *sid2* at later infection stages, indicating a negative-modulatory action of an activated SA pathway on the levels of free, bioactive NHP. At the same time, the accumulation of the presumably inactive glucoside NHPG is markedly attenuated in both *sid2* and *npr1* mutant plants and thus depends on an intact SA signalling pathway (**Figure 4B**; **Supplementary Figure 6**; Hartmann et al., 2018; Bauer et al., 2021; Yildiz et al., 2021). The negative-regulatory influence of the SA pathway on the levels of free NHP might be explained by an NPR1-dependent transcriptional regulation of the NHP glucosyltransferase UGT76B1 (Liu et al., 2020; Bauer et al., 2021). Therefore, depending on the infection stages of *Psm*-inoculated Arabidopsis leaves, SA signalling affects the levels of the SAR-inducer NHP biosynthesis differently: in the early infection stage during which NHP levels only start to rise and are quantitatively low, SA promotes NHP accumulation, while at later times of infection, SA acts as a negative modulator that keeps the quantitatively high levels of NHP that accumulate in these periods under control.

On the resistance level, the direct comparison of *sid2 ald1* and *sid2 fmo1* double mutants with the respective single mutants and the wild-type indicate that SA and NHP signalling add up to guarantee full basal resistance to *Psm* (**Figure 5A**; Bernsdorff et al., 2016). Similarly, additive contributions of SA and NHP were observed with respect to basal resistance of Arabidopsis Col-0 to the compatible bacterial strain *Pst* DC3000 (**Figure 5B**; Liu et al., 2020), and to the compatible *Hpa* isolate Noco2 (**Figure 5C**). These additive effects might either rely on partially independent SA- and NHP-triggered resistance responses that sum up for full basal resistance. Alternatively, they might be based on the mutual enhancement of the SA and NHP defense pathways. For example, previous work has shown that NHP is able to boost the induction of *PR1* expression by SA, while the NHP-triggered priming of Arabidopsis defenses to *P. syringae* challenge was amplified by a functional SA signalling pathway (Yildiz et al., 2021).

Additive genetic contributions of *SID2* and *FMO1* have been previously described also for interactions of Arabidopsis with oomycete or bacterial pathogens that result in ETI. For example, while ETI-based resistance of Col-0 plants to the *H. parasitica* isolate Cala2, which is triggered via the RPP2 resistance gene, was attenuated in both *sid2* and *fmo1*, a *sid2 fmo1* double mutant displayed significantly greater loss of resistance than either *sid2* or *fmo1* alone (Bartsch et al., 2006). Similarly additive contributions of functional *SID2* and *FMO1* were observed for the ETI-related resistance of Col-0 to *Pst AvrRps4* and *Pst AvrRpt2*, which are based on recognition via the RPS4 and RPS2 resistance genes, respectively (Liu et al., 2020). In the current study, the *sid2 fmo1* and *sid2 ald1* double mutants, which were both metabolically characterized for their simultaneous defects in NHP- and SA-biosynthesis (**Figure 1**), showed a higher increase in susceptibility to *Pst avrRpt2* inoculation than *sid2* (**Figure 6A**), corroborating additive contributions of SA and NHP to RPS2-mediated ETI. By contrast, NHP apparently did not contribute to the RPM1-mediated ETI of Arabidopsis to *Psm avrRpm1*, since mutations in *ald1* or *fmo1*, either in the Col-0 or the *sid2* backgrounds, had no negative impact on resistance, although plants harbouring *sid2* mutations showed markedly enhanced susceptibility (**Figure 6A**). These results are consistent with recent findings indicating that a *sid2 ald1* double mutant is more susceptible than each of the single mutants to *Psm* carrying *AvrRpt2*, but not to *Psm* expressing *AvrRpm1* (Yoo et al., 2022). Together, this illustrates that NHP contributes to several but not to all of the distinct resistance gene-mediated ETI responses, while the commonly observed enhanced susceptibility phenotypes of *sid2* corroborate the generally important role of the SA pathway in different forms of ETI. Nevertheless, by providing either indirect genetic data on the function of *FMO1* or direct evidence at the metabolic level, several studies indicate a positive regulatory role of NHP in the execution of ETI-induced or otherwise elicited hypersensitive cell death responses (Olszak et al., 2006; Chen et al., 2018; Hartmann et al., 2018; Czarnocka et al., 2020; Cai et al., 2021).

### SA and NHP additively contribute to the local flagellin-induced acquired resistance response, which exhibits mechanistic similarities and differences to SAR

Flg22-treatment induces a strong acquired resistance response to subsequent bacterial infection in the treated plant tissue (Zipfel et al., 2004; Tsuda et al., 2009). This locally observed acquired immunity should be clearly distinguished from the inducible PTI-response caused by flagellin perception within a running bacterial infection. As reported previously, flg22-triggered acquired resistance entirely depended on a functional flagellin receptor gene FLS2 (**Figure 7A**; Zipfel et al., 2004). Analyses of Arabidopsis mutants impaired in distinct defense pathways also showed that SA signaling significantly contributes to flagellin-induced acquired resistance (Zipfel et al., 2004; Tsuda et al., 2009), which was confirmed in our analyses by the findings that both SA-induction-deficient *sid2* and the SA-insensitive *npr1* mutant showed an attenuated immune response upon flg22-treatment (**Figures 7B-D**). The current study focused on the role of NHP signaling in this context, and our results show that all mutant lines with exclusive defects in the NHP biosynthetic pathway (*ald1, fmo1, ald1 fmo1*) exhibit smaller acquired resistance than the wild-type but more pronounced acquired resistance than *sid2* upon flagellin treatment (**Figures 7A, C**). Therefore, NHP contributes to the locally assessed flagellin-induced acquired resistance to some extent, but this contribution is smaller than the contribution of SA. This is, on a quantitative basis, different to the SAR response induced in systemic tissue by a localized bacterial inoculation. SAR is entirely compromised in all of the mutants unable to accumulate NHP, indicating that NHP acts as an indispensable switch for this systemic response (**Figures 4**; **8A**; Song et al., 2004; Mishina and Zeier, 2006; Bernsdorff et al., 2016; Hartmann et al., 2018). By comparison, SAR induced by bacterial inoculation or NHP treatment is strongly attenuated but not entirely abrogated in *sid2*, emphasizing the importance of SA for SAR establishment on one hand, but also the existence of a small SA-independent SAR-inducing pathway on the other hand (**Figure 8A**; Bernsdorff et al., 2016; Hartmann et al., 2018; Yildiz et al., 2021). Together, this is in line with the notion that SA functions as an amplifier of the NHP-triggered SAR response (Zeier, 2021).

Analyses of the *sid2 ald1* and *sid2 fmo1* double mutants reveal additive contributions of the SA and NHP signaling pathways to flagellin-induced acquired resistance (**Figures 7B, C** **)**, just as it was observed for basal immunity (**Figure 5**). For their immune functions, both SA and NHP require functional NPR1, which is exemplified by the loss of resistance induction by exogenous SA and NHP in *npr1* plants, and the full SAR defect of *npr1* mutants (**Figure 8A**; Feys et al., 2001; Hartmann et al., 2018; Liu et al., 2020; Yildiz et al., 2021). Moreover, similar to the *sid2 ald1* or *sid2 fmo1* double mutants, the flg22-induced immunity was more strongly compromised in *npr1* than in *sid2*, suggesting that SA- and NHP-signaling converge at NPR1 to mediate acquired resistance by flagellin. However, a main difference between local flagellin-induced acquired resistance and SAR is the existence of a significant residual acquired resistance response in the NHP- and SA-deficient double mutants, while SAR is fully absent in these lines (**Figures 7B, C**; **8A**). Therefore, SA- and NHP-independent signaling pathways contribute to flagellin-induced acquired resistance but not to SAR. Flagellin perception activates MAPK cascades that involve MPK3 and MPK6 (Asai et al., 2002; Tsuda et al., 2009; Frei dit Frey et al., 2014). The here-observed wild-type-like flg22-triggered resistance of *mpk3* and *mpk6* either suggest that these MAPKs do not provide significant contributions to flagellin-induced acquired resistance against *P. syringae* challenge, or that *MPK3* and *MPK6* act redundantly in this process (**Figure 7C**). However, a previous study observed a compromised and attenuated flg22-induced resistance to *Botrytis cinerea* infection in *mpk6* and *mpk3* plants, respectively, indicating that flagellin responses to other pathogens might develop via activated MPK3/6 signaling (Galletti et al., 2011). Moreover, *MPK3* and *MPK6* participate in the flagellin-induced suppression of ETI responses (Wang et al., 2023).

We also observed that flagellin-induced resistance and SAR develop to lower absolute levels in *pad4* and *eds1* than in the wild-type, which is consistent with previous findings that *PAD4* contributes to flg22-induced immunity (Tsuda et al., 2009). PAD4 associates with EDS1 to mediate pattern-triggered immunity (Pruitt et al., 2021), explaining that flagellin-induced immunity was attenuated to similar levels in *pad4* and *eds1* plants in our analyses (**Figure 7C**). Following pathogen inoculation, PAD4 and EDS1 positively regulate both SA and NHP biosynthesis (Feys et al., 2001; Hartmann et al., 2018), which could explain the observed attenuated flagellin and SAR responses in the respective mutants (**Figures 7C**, **8B**). Interestingly, a quadrupole mutant defective in *PAD4*, *SID2*, the jasmonic acid (JA) biosynthesis gene *DDE2* and the ethylene (ET) signaling gene *EIN2* display a markedly greater loss of flagellin-induced resistance to *P. syringae* than *pad4* or *sid2* mutants, indicating contributions of both JA and ET signaling (Tsuda et al., 2009). Whether JA- and ET-mediated signaling trigger the above-described SA- and NHP-independent resistance pathways that induce the residual flagellin-induced acquired resistance in *sid2 ald1* and *sid2 fmo1* double mutants remains to be examined.

We have previously observed that flg22-treatment of single Arabidopsis leaves induces a moderate immune response also in the distant leaves (Mishina and Zeier, 2007). This systemic resistance response to flagellin was entirely absent in *fmo1*, *sid2* and *npr1* mutants and thus closely resembles the SAR response triggered systemically by an inducing pathogen inoculation. It is important to note that the local and not the systemic acquired response to flagellin was investigated in the present study.

### The resistance phenotypes of ald1 fmo1 plants argue against NHP-independent functions of Pip, ALD1 and FMO1 in plant immunity

The importance of the pipecolate pathway in SAR was first described in a study by Návarová et al. (2012), which identified a critical role of *ALD1*-dependent Pip accumulation in SAR and demonstrated that exogenous Pip triggers a resistance response reminiscent to SAR in Arabidopsis. However, both the transcriptional and resistance response associated with Pip-induced SAR entirely depended on the function of the critical SAR gene *FMO1* (Mishina and Zeier, 2006; Návarová et al., 2012; Bernsdorff et al., 2016; Chen et al., 2018; Hartmann et al., 2018), suggesting that an FMO1- and Pip-derived metabolite acts as a triggering signal for SAR. These findings were followed by unequivocal biochemical characterization of FMO1 as an NHP synthase that N-hydroxylates Pip to generate NHP, both *in vitro* by purified FMO1 (Hartmann et al., 2018), in Arabidopsis plants by use of isotope-labelled Pip (Hartmann et al., 2018), in *Nicotiana benthamiana* transiently expressing Arabidopsis *FMO1* (Chen et al., 2018), and in transgenic tomato or tobacco in which the three Arabidopsis NHP biosynthetic genes *ALD1*, *SARD4*, and *FMO1* were concomitantly expressed (Holmes et al., 2019; Cai et al., 2021). NHP potently induced systemic immunity in Arabidopsis and other mono- and dicotyledonous plants, and NHP but not Pip was able to rescue SAR in the NHP-deficient *fmo1* mutant (Chen et al., 2018; Hartmann et al., 2018; Holmes et al., 2019; Schnake et al., 2020; Yildiz et al., 2021). These distinct biochemical and physiological lines of evidence demonstrate that NHP is the immune-active, SAR-inducing metabolite and Pip “merely” functions as the necessary biosynthetic precursor of NHP. This perspective is corroborated by the fact that SAR is inactivated at the level of NHP glycosylation, which was reported independently by several research studies (Bauer et al., 2021; Cai et al., 2021; Holmes et al., 2021; Mohnike et al., 2021; Zeier, 2021). Nevertheless, some recent studies that functionally investigated immune responses genetically at the level of *ALD1* or by Pip application center Pip in their SAR-related working models (e.g., Wang et al., 2018b; Jiang et al., 2021). In the present study, we have directly compared a number of different resistance responses of Pip-accumulating but NHP-deficient *fmo1* with Pip- and NHP-deficient *ald1* and *ald1 fmo1* in which functional FMO1 was either present (*ald1*) or absent (*ald1 fmo1*) (**Figure 4**). For all the immune types and defense reactions tested – i.e., basal resistance to different bacterial and oomycete pathogens (**Figure 5**), SA and camalexin accumulation associated with *Psm*-triggered responses (**Figure 4**; **Supplementary Figure 5**), ETI to avirulent bacterial pathogens (**Figure 6**), flagellin-triggered acquired resistance (**Figure 7**), and *P. syringae*-induced SAR (**Figure 8A**), the responses of *ald1*, *fmo1* and *ald1 fmo1* were similar. These findings further corroborate the above-mentioned reasoning that Pip functions as an important direct precursor of the immune-active FMO1-product NHP, and argue against an autonomous resistance-enhancing activity of Pip. Moreover, they argue against FMO1-related immune functions beyond the NHP pathway.

The full SAR defects of *ald1*, *fmo1*, and *ald1fmo1* together with the thoroughly characterized NHP biochemical pathway demonstrate the necessity of NHP accumulation in plants for the biological induction of SAR (**Figure 4**; Návarová et al., 2012; Bernsdorff et al., 2016; Ding et al., 2016; Hartmann et al., 2017; Chen et al., 2018; Hartmann et al., 2018). In this light, reports on resistance-enhancing activites of petiole exudates from NHP-deficient *ald1* plants appear physiologically irrelevant for biological SAR (Yoo et al., 2022). With respect to the function of NHP in long-distance signaling, two possible scenarios are conceivable: 1) NHP accumulating in inoculated leaves is partly mobilized to distant leaves in which it activates SAR (Chen et al., 2018; Cai et al., 2021; Mohnike et al., 2021; Yildiz et al., 2021; Yildiz et al., 2023). And 2), enhanced NHP levels induce molecular and cellular processes in the inoculated leaves that mediate signal propagation to distant leaves. A contribution of the second scenario is suggested by the resistance phenotype of transgenic Arabidopsis plants in which *ALD1* was specifically expressed in epidermal tissue (Jiang et al., 2021). The hitherto conducted studies indicate that a combination of both scenarios orchestrate SAR long-distance signaling (Zeier, 2021).

## Data availability statement

The original contributions presented in the study are included in the article/**Supplementary Material**. Further inquiries can be directed to the corresponding author.

## Author contributions

ML, KJ, TZ, MH, KG, SM, IY, and MP performed the experiments and analysed data, JZ conceived and designed the experiments, analysed data and wrote the manuscript. All authors contributed to the article and approved the submitted version.
