# Supplementary material for: N-hydroxypipecolic acid primes plants for enhanced microbial pattern-induced responses
Source: Front Plant Sci. 2023 Aug 14;14:1217771. doi: 10.3389/fpls.2023.1217771 (PMC10461098; doi:10.3389/fpls.2023.1217771)
Supplement: Supplementary file 1 [file DataSheet_1.pdf]

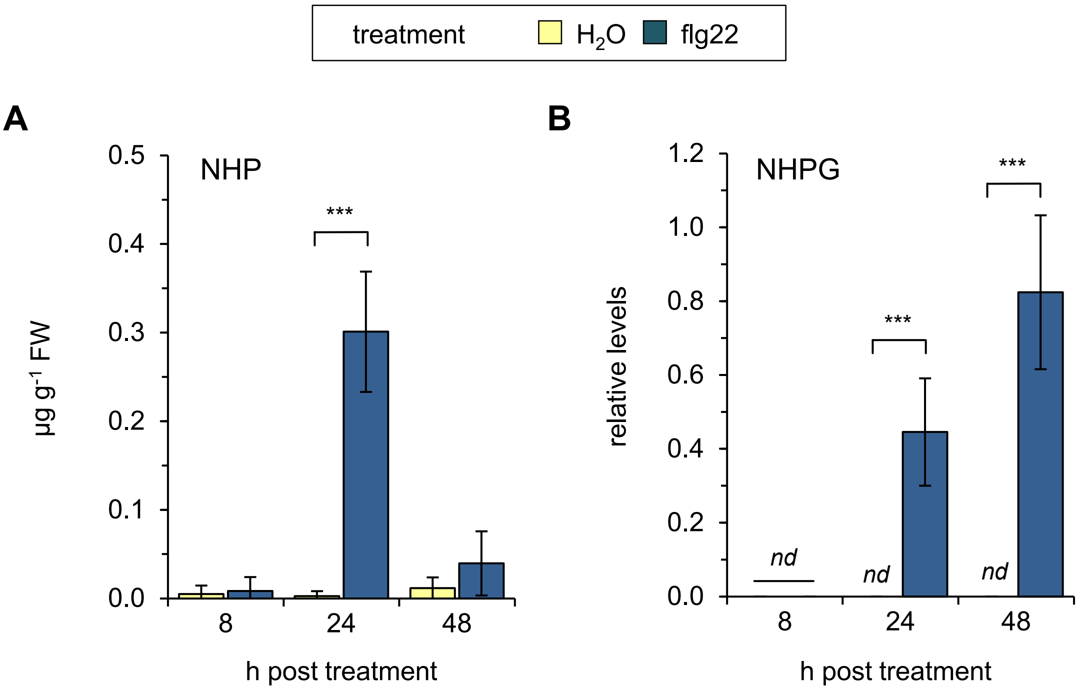

Supplementary Figure 1 Treatment with flg22 peptide triggers accumulation of NHP and the glucoside NHPG in Arabidopsis Col-0 leaves. Leaves of plants were infiltrated with 1 µM flg22 (flg) or water (w). Metabolite levels in leaves were determined 8, 24, and 48 h after treatments. (A) Levels of N-hydroxypipecolic acid (NHP) in µg g^{-1} fresh weight (FW). (B) Levels of NHP-β-glucoside (NHPG) are given as relative, FW-related amounts. Bars represent means ± SD of five to six biological replicates. *nd*: not detected.

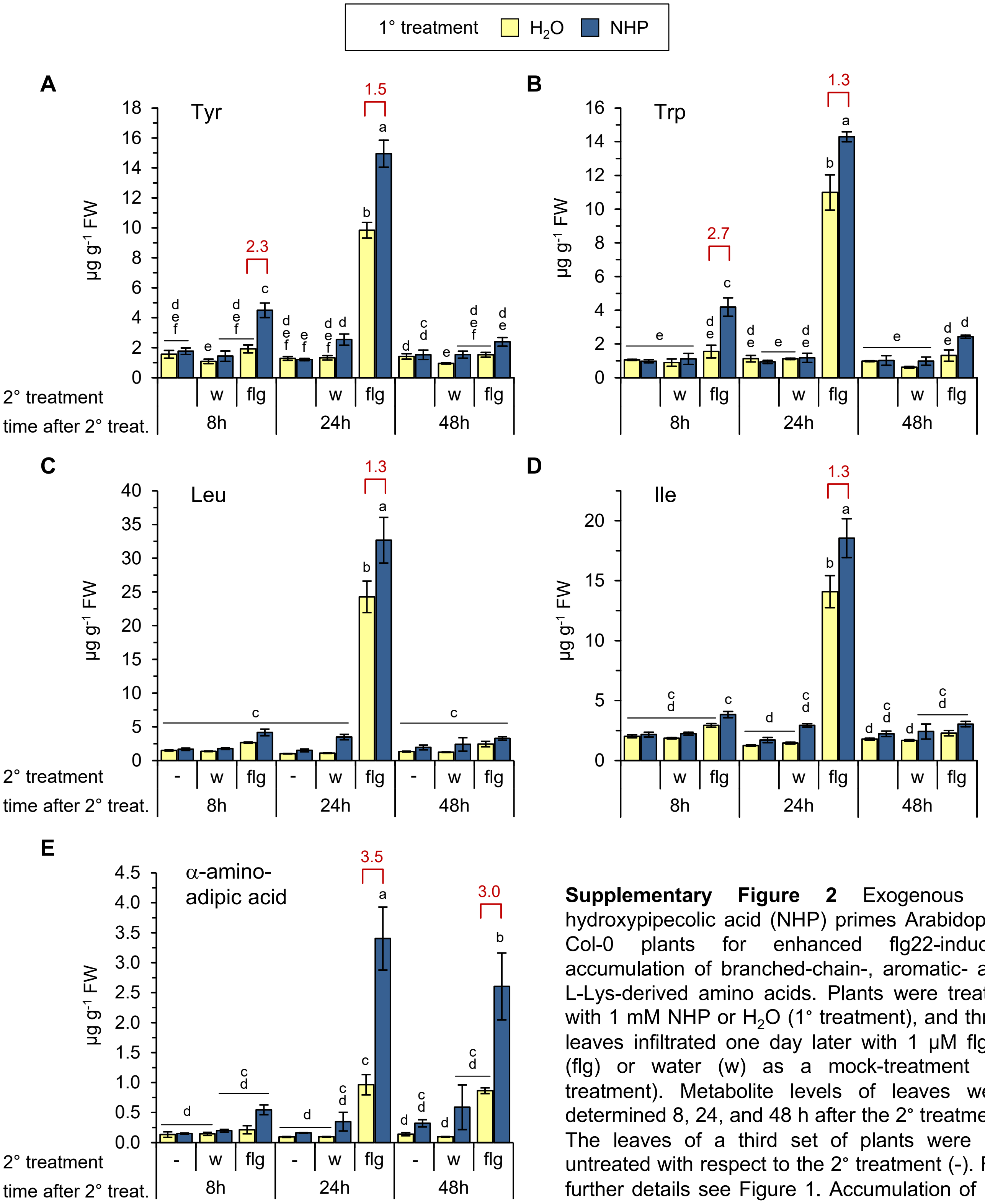

Supplementary Figure 2 Exogenous N-hydroxypipecolic acid (NHP) primes Arabidopsis Col-0 plants for enhanced flg22-induced accumulation of branched-chain-, aromatic- and L-Lys-derived amino acids. Plants were treated with 1 mM NHP or H_2O (1° treatment), and three leaves infiltrated one day later with 1 µM flg22 (flg) or water (w) as a mock-treatment (2° treatment). Metabolite levels of leaves were determined 8, 24, and 48 h after the 2° treatment. The leaves of a third set of plants were left untreated with respect to the 2° treatment (-). For further details see Figure 1. Accumulation of (A) tyrosine, (B) tryptophan, (C) leucine, (D) isoleucine, and (E) α-amino adipic acid [in µg g^{-1} fresh weight (FW)]. Bars represent means ± SD of three biological replicates (n = 3). Different letters denote significant differences ($p < 0.05$, ANOVA and post-hoc Tukey HSD test). The degree of priming of flg22-responses is illustrated by a priming factor (red values; see Figure 1).

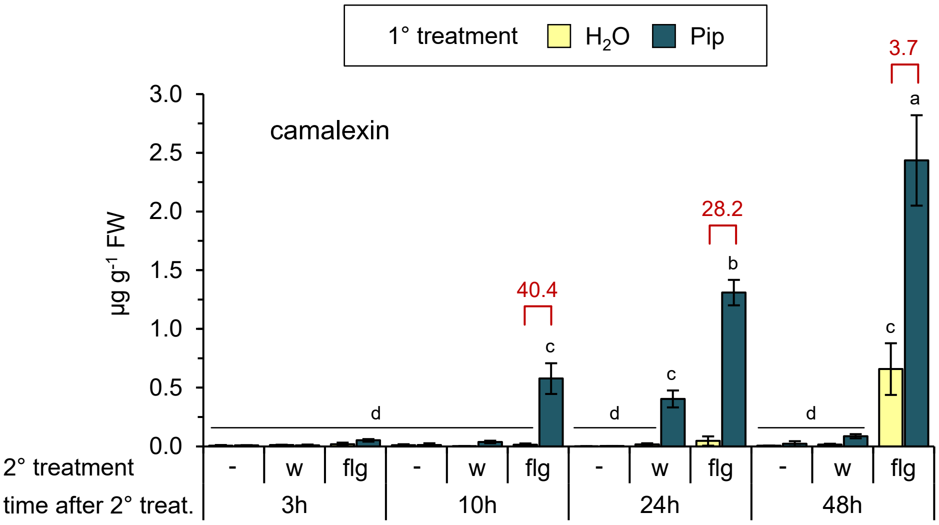

Supplementary Figure 3 Exogenous application of the NHP precursor pipecolic acid (Pip) primes Arabidopsis for enhanced flg22-induced accumulation of camalexin. Plants were treated with 1 mM Pip or H_2O (1° treatment), and three leaves infiltrated one day later with 1 µM flg22 (flg) or water (w) as a mock-treatment (2° treatment). Leaf camalexin levels were determined 3, 10, 24, and 48 h after the 2° treatment. The leaves of a third set of plants were left untreated with respect to the 2° treatment (-). Camalexin levels are given in µg g^{-1} fresh weight. Bars represent means ± SD of three biological replicates (n = 3). Different letters denote significant differences ($p < 0.05$, ANOVA and post-hoc Tukey HSD test). The degree of priming of flg22-responses is illustrated by a priming factor (red values; see Figure 1).

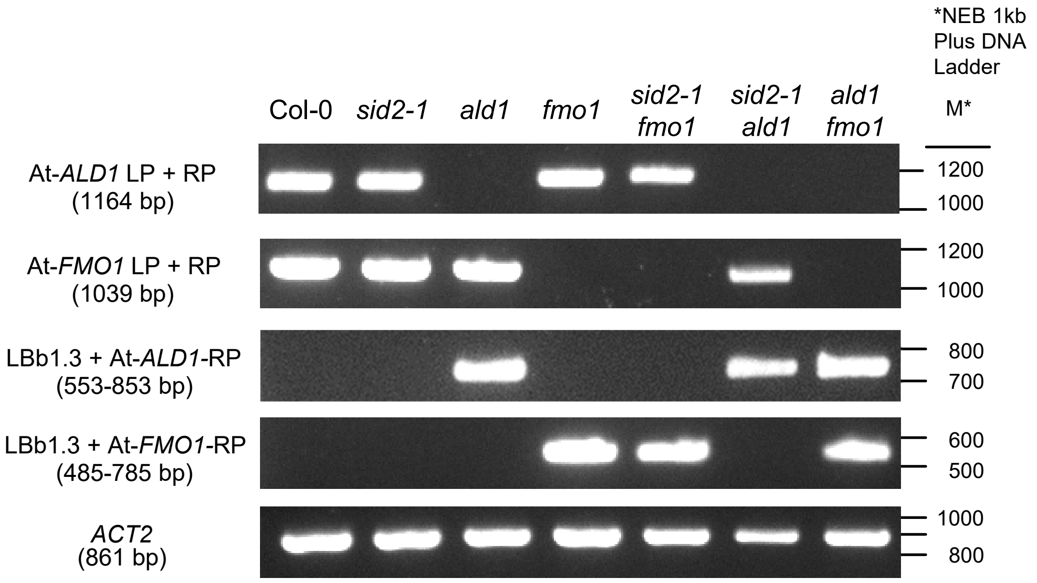

Supplementary Figure 4 PCR-based genotyping to characterize *sid2-1 fmo1* and *ald1 fmo1* double mutants. The results of PCR analyses of genomic DNA from Col-0 wild-type, *sid2-1*, *ald1*, *fmo1*, *sid2-1 fmo1*, *sid2-1 ald1*, and *ald1 fmo1* plants are shown for comparison. The characterization of *sid2-1* (EMS-mutant), *ald1* (Salk_007673), *fmo1* (Salk_026163), and *sid2-1 ald1* lines was reported previously (Mishina and Zeier, 2006; Nawrath and Métraux, 1999; Návarová et al., 2012; Bernsdorff et al., 2016). Gene specific forward (LP) and reverse (RP) primers for *ALD1*, *FMO1*, and actin (*ACT2*) and the specific left border primers LBb1.3 T-DNA insertion lines of the Salk collection were used in the PCR reactions, and amplified bands in a DNA gel electrophoresis are shown. The position of DNA ladder bands is indicated on the right, and expected band sizes or ranges of sizes are shown on the left (Brackets). For the identification of *sid2-1 fmo1* double mutants, plants were screened for SA-induction-deficiency upon *Psm* inoculation in parallel (see Figure 4).

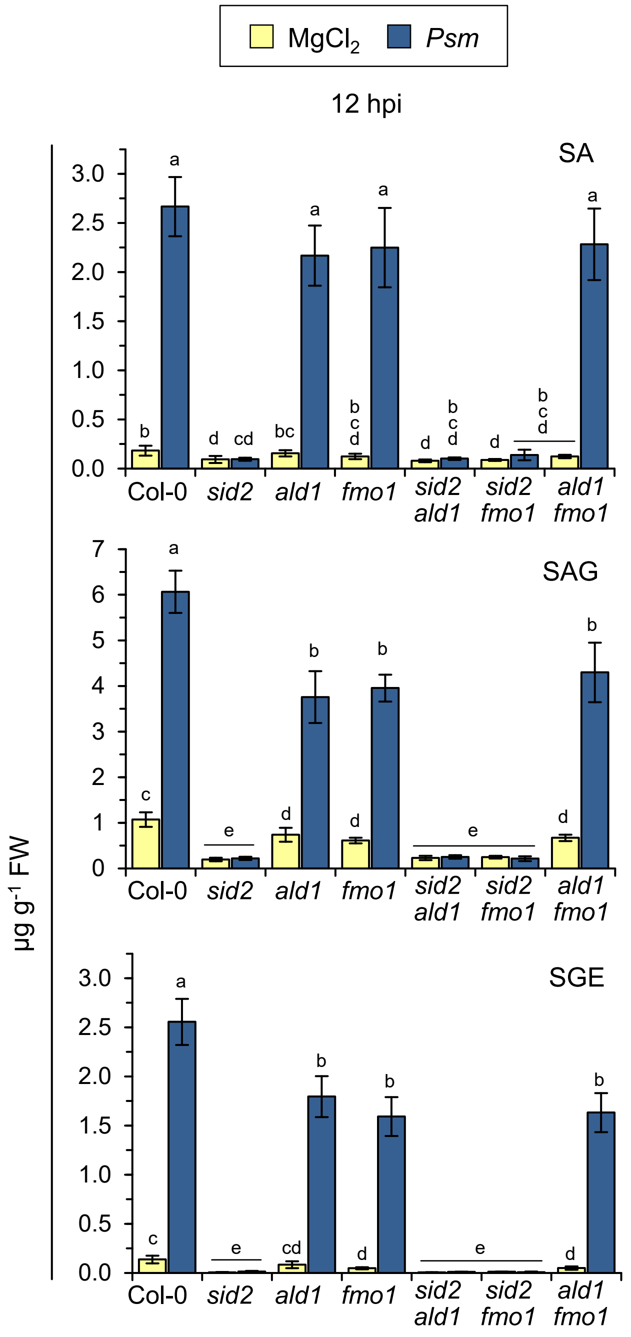

Supplementary Figure 5 Accumulation of unconjugated salicylic acid (SA), SA-β-glucoside (SAG) and SA glucose ester (SGE) in Arabidopsis wild-type plants and mutant lines defective in NHP- and/or SA-biosynthesis at 12 h post bacterial inoculation (hpi). Plants were inoculated with compatible *P. syringae* pv. *maculicola* (*Psm*) or mock-treated with 10 mM $MgCl_2$. Bars represent means ± SD of five biological replicates (n = 5). Different letters denote significant differences ($p < 0.05$, Kruskal-Wallis H test). *nd*: not detected. Related to Figure 4A.

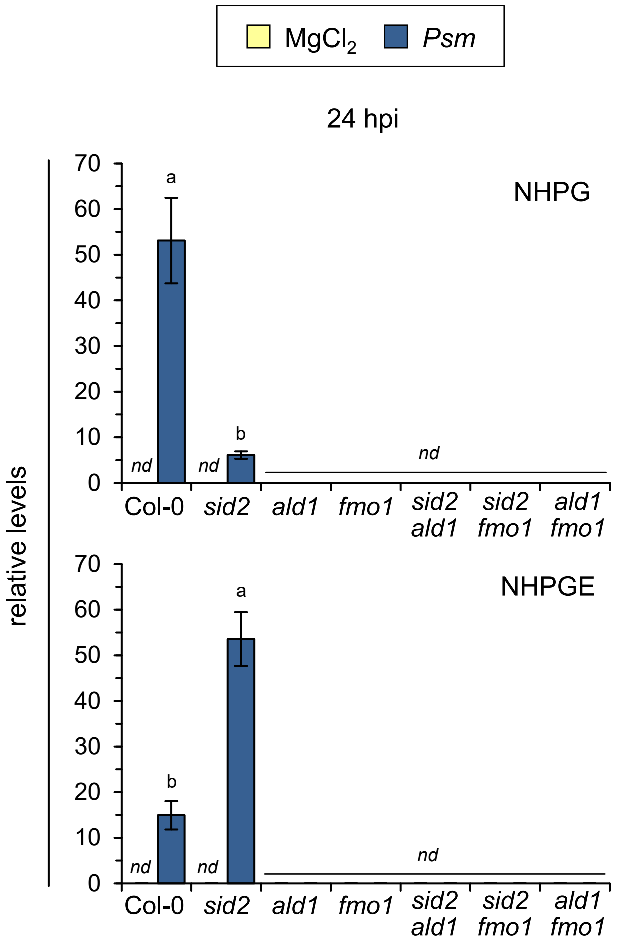

Supplementary Figure 6 Accumulation of N-hydroxypipecolic acid (NHP)-β-glucoside (NHPG) and NHP glucose ester (NHPGE) in Arabidopsis wild-type plants and mutant lines defective in NHP- and/or SA-biosynthesis at 24 h post bacterial inoculation (hpi). Plants were inoculated with compatible *P. syringae* pv. *maculicola* (*Psm*) or mock-treated with 10 mM $MgCl_2$. The levels of NHPG and NHPGE are given as relative, fresh weight-related levels, since authentic standards necessary for absolute quantification were unavailable (see methods). Bars represent means ± SD of four biological replicates (n = 4). Different letters denote significant differences ($p < 0.05$, Kruskal-Wallis H test). *nd*: not detected. Related to Figure 4B.

Primer name	Primer sequence (5’ to 3’)	Usage
At-FMO1 LP	CTTTTCGGTTGGACTTGGAAC	Left primer; genotyping of *sid2-1 fmo1* and *ald1 fmo1*
At-FMO1 RP	CTGCTTTGGACGTATCCTACG	Right primer; genotyping of *sid2-1 fmo1* and *ald1 fmo1*
At-ALD1 LP	TTACGATGCATTTGCTATGACC	Left primer; genotyping of *ald1 fmo1*
At-ALD1 RP	TTTTAAATGGAACGCAAGGAG	Right primer; genotyping of *ald1 fmo1*
LBb1.3	ATTTTGCCGATTTCGGAAC	T-DNA Left Border primer (Salk lines)
ACT2 LP	TCGCCATCCAAGCTGTTCTCT	genotyping
ACT2 RP	CCTGGACCTGCCTCATCATACTC	genotyping
PAD3-FW	GGCTGAAGCGGTCATAAGAG	qRT-PCR
PAD3-RW	TCCAGGCTTAAGATGCTCGT	qRT-PCR
ALD1-FW	GTGCAAGATCCTACCTTCCCGGC	qRT-PCR
ALD1-RV	CGGTCCTTGGGGTCATAGCCAGA	qRT-PCR
FMO1-FW	TCTTCTGCGTGCCGTAGTTTC	qRT-PCR
FMO1-RV	CGCCATTTGACAAGAAGCATAG	qRT-PCR
ICS1-FV	TTCTGGGCTCAAACACTAAAAC	qRT-PCR
ICS1-RV	GGCGTCTTGAAATCTCCATC	qRT-PCR
PBS3-FW	TGCCTGCTCGAGTCGCAACC	qRT-PCR
PBS3-RV	TGGACTAAGCCACAGAGCAAATGGC	qRT-PCR
FRK1-FV	ACAACAAAGAGGTACACTTGG	qRT-PCR
FRK1-RV	CTTGTTCTCCATTTATGACACC	qRT-PCR
PTB-FW	GATCTGAATGTTAAGGCTTTTAGCG	qRT-PCR; reference gene
PTB-RV	GGCTTAGATCAGGAAGTGTATAGTCTCTG	qRT-PCR; reference gene

Supplementary Table 1 List of primers used in this study.
